# Nature-inspired solutions for energy sustainability using novel optimization methods

**DOI:** 10.1371/journal.pone.0288490

**Published:** 2023-11-27

**Authors:** Abdulwahab Ali Almazroi, Ch Anwar Ul Hassan

**Affiliations:** 1 Department of Information Technology, College of Computing and Information Technology at Khulais, Univeristy of Jeddah, Jeddah, Saudi Arabia; 2 Department of Creative Technologies, Air University, Islamabad, Pakistan; King Fahd University of Petroleum and Minerals, SAUDI ARABIA

## Abstract

This research centres on developing a Home Electricity Management (HEM) system, a pivotal component within the modern supply chain for home electrical power. The system optimizes the scheduling of intelligent home gadgets through advanced meta-heuristics, specifically the Social Spider Algorithm (SSA) and Strawberry Algorithm (SWA), to efficiently manage home energy consumption. Within the supply chain context, HEM acts as a crucial link in the distribution and utilization of electricity within households, akin to optimizing resource allocation and demand balancing within a supply chain for efficient operation and cost-effectiveness. Simulations and comparisons demonstrate that SWA excels in cost savings, while SSA is more effective in reducing peak-to-average power ratios. The proposed solution reduces costs for residences by up to 3.5 percent, highlighting the potential for significant cost savings and efficiency improvements within the home electricity supply chain. It also surpasses existing cost and Peak Average (PAR) ratio meta-heuristics, indicating superior performance within the overall energy supply and consumption framework. Moreover, implementing the HEM system contributes to reducing carbon emissions, aligning with sustainability goals in the energy supply chain. It promotes energy efficiency, integrates renewable sources, and facilitates demand response, mirroring the emphasis on sustainability in supply chain practices. Overall, this research offers a practical and sustainable approach to home energy management, bringing substantial cost savings and environmental benefits to the modern supply chain for residential electricity.

## Introduction

The resource that fuels our contemporary world is electricity. The need for power keeps rising as economies and populations worldwide expand. Nevertheless, such enormous demands were not intended for our conventional power systems. As a result, our power networks frequently experience overload and are vulnerable to blackouts, which can seriously affect the economy and society [1]. In this context, the integration of a Home Electricity Management (HEM) system, as discussed in the earlier abstract, becomes increasingly crucial within the broader context of the supply chain for electricity management in residential settings.

A Smart Grid (SG), a more effective and dependable alternative to conventional electricity systems, has been suggested to address the challenges associated with the increasing demand for power. The integration of cutting-edge technologies and communication systems into our electrical infrastructures is essential for managing the supply and demand of electricity within this evolving landscape. The implementation of the Home Electricity Management (HEM) system serves as an essential component in realizing the objectives of a Smart Grid by optimizing energy consumption within households.

Furthermore, the use of smart appliances and smart meters, as integral components of the Smart Grid, facilitates the efficient management of electrical grids while providing consumers with precise and real-time information about their energy usage [2]. The interplay between the HEM system and the Smart Grid is crucial in ensuring seamless coordination between residential energy consumption patterns and the broader demands and fluctuations within the larger grid system.

Through the integration of diverse energy sources and the incorporation of energy-saving tools such as demand response plans and energy storage systems [3], the Smart Grid can ensure the reliability and stability of the overall energy supply chain. The implementation of the HEM system aligns with these objectives, contributing to efficient energy management within households and actively participating in the optimization of the broader electricity supply chain infrastructure.

SM acts as a channel for utility companies and consumers to exchange information. Using this information, smart houses’ energy efficiency is then optimized. According to the available literature, there are various approaches recommended for efficiently controlling power in a smart grid through demand-side management (DSM). These methods include strategically scheduling electric appliances in homes, managing power distribution, and implementing nature-inspired techniques to optimize energy resource management within the smart grid, thus enhancing overall efficiency. By managing power demand and facilitating a dialogue between energy providers and consumers, with a primary focus on power optimization, the smart grid (SG) functions as a bidirectional or mutual communication device. This improved information and communication enables a more beneficial energy cohort and circulation. Consumers can optimize their energy use patterns using the Energy Management System (EMS) using load shifting, peak clipping, and valley filling strategies. The implementation of this step-by-step approach helps achieve stability in both supply and demand. These tactics encourage individuals to relocate their high-load consumption times to out-peak periods. Demand and supply are the two primary streams of DSM regarding response and control [4].

Effective management of a user load profile is the aim of load management. Because the strain on the power system has been alleviated and blackouts have been avoided, the stability of the electrical grid is maintained. Successful management of energy efficiency entails reducing the Peak to Average Ratio (PAR), power usage, and utility prices. The author of [5] asserts that despite being characterized as a user’s response to the fluctuating pricing rates set by a utility, addressing concerns about grid stability is becoming more difficult due to the significant surge in energy consumption. The imbalance between demand and supply patterns arises when power consumers’ load demands increase, leading to load shedding that jeopardizes the grid’s stability across a significant area. DR is, therefore, an efficient method of persuading energy consumers to alter their usage habits in exchange for incentives and minor inconvenience. DR programmers employ two sorts of strategies: pricing and incentive techniques. Technique based on Price DR includes Dynamic Pricing systems (DP), where flat-rate pricing schemes establish a more accurate demand-supply relationship than DP pricing approaches. Time of Use (TOU), Day-Ahead Pricing (DAP), Critical Peak Pricing (CPP), Inclined Block Rate (IBR), and Real-Time Pricing (RTP) are some of the available DP tariffs, with RTP being the most It is the effective pricing strategy for electricity markets under the above approach [6].

HEM implemented through optimization methods offer a promising solution to reduce carbon emissions associated with residential energy consumption. By employing intelligent algorithms and advanced optimization techniques, HEMS enable homeowners to actively manage and optimize their energy usage, leading to significant reductions in carbon emissions. Load scheduling, in which appliances are intelligently planned to function during off-peak hours or when renewable energy sources are more plentiful, is a crucial component of home energy management. By strategically shifting energy-intensive tasks and reducing peak-hour loads, HEMS can alleviate stress on the grid and minimize the reliance on fossil fuel-based power generation, consequently reducing carbon emissions. Furthermore, optimization methods within HEMS enable the efficient utilization of renewable energy resources. By integrating data on renewable energy availability, weather conditions, and household energy demand, HEMS algorithms can dynamically allocate energy from renewable sources to power appliances and charge energy storage systems. By maximizing sustainable energy sources, this optimization lessens the need for non-renewable energy and lowers carbon emissions. HEMS may also give useful information about energy usage trends, allowing homeowners to choose energy-saving solutions with confidence. In the realm of home energy management, sustainable practices are facilitated and carbon footprints are reduced through the utilization of optimization methods. Energy use is tracked, energy-hungry equipment is detected, and feedback on energy-saving solutions is provided, enabling individuals to adopt sustainable practices and lower their carbon footprint. In the broader context, the application of optimization methods in home energy management provides a comprehensive approach to minimize carbon emissions associated with residential energy consumption. Load scheduling is optimized, renewable energy sources are leveraged, and energy-efficient behaviors are promoted, all contributing to a greener and more sustainable future. This alignment with global efforts to combat climate change and reduce greenhouse gas emissions underscores the significance of home energy management in achieving environmental goals [6, 7].

The shared objectives are to reduce power prices, improve user comfort, maximize PAR, reduce aggregate power usage, and incorporate renewable energy sources. Several DSM tactics have emerged in recent years to achieve the aforementioned objectives. To save money and energy, use optimization techniques, including non-linear mixed-integer programming, convex programming, fractional programming, and linear mixed-integer programming [7, 8]. Nevertheless, these approaches are inadequate for handling a significant collection of gadgets. Numerous metaheuristic optimization approaches for energy administration concepts are presented as a result of the limitations of the aforementioned methods. The authors of [9, 10], for instance, employed a genetic algorithm (GA) to save electricity expenses, Use differential evolution (DE) to reduce electricity prices and accumulated power consumption in addition to Ant Colony (AC) in [11].

The main objectives of this study are as follows:

- To develop an innovative hybrid scheduling approach by combining the Social Spider Algorithm (SSA) and the Strawberry Algorithm (SWA).
- To optimize the scheduling of smart home devices in a way that minimizes energy costs while maintaining user comfort.
- To reduce the peak-to-average ratio (PAR) in energy consumption, which benefits both consumers and utility providers by enhancing grid stability and reducing costs.
- To minimize carbon emissions by scheduling the operation of smart home devices more efficiently.

By achieving these objectives, this research aims to provide a comprehensive and novel solution for smart home device scheduling that offers cost savings, reduced energy consumption peaks, enhanced user comfort, and environmental benefits.

The present investigation explored the effectiveness of dynamic pricing in motivating manipulators to shift their loads from maximum load prime hours. We applied meta-heuristic techniques to lower the PAR, improve the consumer’s facility, and reduce power usage. This paper employs metaheuristic—methods. Hybrid SSWA (SSA and SWA) algorithms have been tested to comprehend how we can monitor and operate smart machines in both multiple and single households, similar to Spider Search (SSA) and Strawberry Algorithm (SWA).

### Contributions and novelty

This study makes several significant contributions to the field of smart home energy management and optimization. Our research introduces innovative approaches and novel findings that address critical challenges in this domain:

1. Hybrid SSA and SWA Algorithm: We propose a novel hybrid algorithm that combines the strengths of the Social Spider Algorithm (SSA) and the Strawberry Algorithm (SWA). This unique fusion leverages the efficient path-finding capabilities of SSA with the robust optimization characteristics of SWA. Our approach, distinct from previous studies, provides an advanced and effective method for scheduling smart home devices.
2. Multi-Objective Optimization: Unlike traditional approaches that primarily focus on a single optimization criterion, our study addresses multiple objectives simultaneously. We optimize not only energy costs but also consider peak-to-average ratio (PAR), user comfort, and carbon emissions. This holistic approach ensures a more comprehensive and balanced solution for smart home energy management.
3. Improved Energy Efficiency: Our research demonstrates a substantial improvement in energy efficiency through the deployment of the hybrid SSA and SWA algorithm. By minimizing energy consumption during peak hours and strategically scheduling device operations, we achieve remarkable cost savings while reducing the overall energy pattern.
4. Enhanced User Comfort: We evaluate user comfort by assessing waiting times for device operation. While it is known that cost-saving strategies often lead to increased waiting times, our approach strikes a balance between cost reduction and user convenience. The proposed algorithm offers flexible scheduling options that minimize waiting times without compromising substantial cost savings.
5. Statistical Analysis: To validate the performance of our hybrid algorithm, we conduct a comprehensive statistical analysis. This analysis provides robust evidence of the superiority of our approach over existing methods in terms of cost savings, energy efficiency, carbon emissions reduction, overall performance, PAR, and user comfort.

Our study’s primary contributions lie in the development of a novel hybrid algorithm, the integration of multi-objective optimization, improved energy efficiency, enhanced user comfort, and the provision of rigorous statistical evidence of our algorithm’s effectiveness. These contributions collectively advance the state-of-the-art in smart home energy management and offer valuable insights for researchers and practitioners in the field.

### Paper organization

The article is divided into many parts. Part 2 provides a historical perspective, and Section 3 provides related work examples. Section 4 deals with the issue. Section 5 describes recommended system models. Section 6 details the approach we used to maximize the energy. In sections 7 and 8, Simulation results are illustrated as desecration of results, respectively. Section 9 presents results and recommendations for further investigation.

## Related work

To significantly improve the SG, many optimization techniques have recently been released. The ideal solution’s two key objectives are load management and cost-cutting cutting. The literature on numerous optimization techniques is now available, and we shall address it in this chapter. The authors of [12] have published an effective Demand Side Management model (DSM) for country regions. They aimed to increase user convenience while reducing execution time, PAR, and energy costs. The author used GA, Binary PSO (BPSO) and ACO to achieve his goal. To set up the electricity tariff, they combined ToU and IBR pricing. An Energy Management Controller (EMC) was utilized in this study to control how much energy is consumed by houses (HEM). Compared with BPSO and ACO, The findings indicate that the suggested GA model works well regarding expenditure devaluation and PAR minimization. GA also provide the fastest response time compared to other optimization strategies. According to the customers’ energy use patterns, the researchers separated the three types of appliances into three categories. The authors of [13] employed an evolutionary approach based on heuristics to address the optimization issue. The study’s objectives were to increase PAR and reduce energy costs. The authors considered users in the private, public, and industrial sectors. The findings show that the recommended algorithm can handle various controlled device types while drastically lowering PAR and power costs. Many contemporary electric appliances are independent and programmed to respond to signals about utility costs. However, peak creation may happen when everyone tries to organize capacity during off-peak hours. The authors use the Multi-Objective Evolutionary (MOE) strategy in [14] to fine-tune power consumption and prevent over- or underutilization of the network. This study’s primary concern is reducing power bills by implementing efficient energy consumption. The delay computation formula is applied to determine the operating time for each appliance while each load is assigned a priority. High-priority devices take precedence over low-priority ones, increasing the delay period of the less important appliance. The average latency of all devices is computed using a pre-established function to diminish typical waiting times for appliances and decrease the expenses related to energy consumption. Similarly, researcher in [15] introduces a cost-saving methodology for SG relying on GA. The RES maintains battery packs and prohibits the battery’s power from being utilized when energy is in high demand or at a high price. Batteries have limitations on their capacity to charge and discharge to prevent damage, and they also include a controller that governs these activities. However, one issue with this study is how rigidly the pressure is applied [16]. To save expenditures on electricity and energy use, there is frequently a controlled load that may be shifted in a household or commercial setting. With batteries charged either by RES or utility, the RTP model optimizes the load while energy prices are low. When power is expensive and renewable energy is used, batteries are emptied to meet demand. Power must be taken from the core system when renewable power and battery resources are inadequate to satisfy a need. This results in lower energy consumption costs and more efficient energy usage. Additionally, it has been discovered that a greater battery volume leads to more efficient power use and lower costs [16].

Researcher in [17], a proficient energy control system is suggested, which prioritizes the management of the supply chain. The study achieves the best configuration values through the utilization of diverse decentralized energy assets. The scheduling process uses the modified bacterial foraging algorithm (MBFO), which lowers operational expenses and net emissions. Nevertheless, this study does not take into account DSM. On the other hand, the authors of [18] suggest a hybrid DE through harmony search (DE-HS) technique. The authors anticipate the age group development of a microgrid that includes old-style generators, photovoltaic (PV) systems, wind energy generation, electric vehicles (EV), and battery storage. DE-HS algorithm addresses scheduling tasks. The researchers address how solar and wind technologies cannot sustain microgrid stability. According to simulation findings, the suggested fusion technique involves little capital. He investigated two alternatives: microgrid (MG) programming with electric vehicles (EVs) and storage systems and MG programming excluding EVs and storage. With a 7.83% cost reduction, the suggested technique performs better in the initial case (with EVs and storage).

A prototype of day-ahead scheduling of MG was provided by the authors in [19] and included a wind turbine (WEA), a photovoltaic system (PV), a diesel generator (DG) and a battery. The authors projected a hybrid methodology HSA with DE (HSDE). This is Fusion GA with DE (GADE), Modified DE (MDE), Hybrid PSO with DE, and fusion of Artificial Immune Algorithm and DE (AIADE) (PSODE). The HSDE method outperformed all other compared techniques regarding superior convergence, including minimal CPU time and low cost. The proposed hybrid technique was improved by incorporating selection, mutation, and competition to enhance its searching capabilities. The resilience of MG was evaluated in both normal and fault mode operation using industry-standard IEEE 57, 39 and 9 bus systems. In [20], turning off appliances with low priority can reduce energy use, expenses, and PAR. Appliances are ranked according to user preferences. In [21], the author compares greedy search and simulated annealing approaches with a GA-based methodology for maximum cutting costs in consumer devices. Although the GA-based model performed better in reducing costs, the study neglected the impact on user comfort. In [22], a DSM model was described based on smart switches and involves assigning priorities to each appliance. A global positioning system accurately determines how each device operates, reducing power usage by 11.2%.

The author of [23] illustrates a proficient HSA for managing the timing of energy storage using sustainable energy sources. The integration of a pricing approach TOU, is coupled with a demand management strategy. The writer investigates the load and generation patterns of household consumers. In [24], the writer introduces an inventive plan for arranging intelligent devices in residential regions, contrasting it with a GA to showcase its efficiency. The findings indicate that HSA outperforms GA. The proposed solutions are assessed utilizing efficient procedures that consider both pricing and processing time. The results reveal that the heuristic technique has a cost under 5% of the estimated cost. Yet, the processing time is decreased in half. In [25], the author introduces an original scheduling framework for domestic devices. Using heuristics and MILP, the authors develop a scheduling model. According to the model findings, the heuristic-based technique is more advantageous regarding efficiency and efficacy. Unfortunately, it disregards PAR, increasing system complexity.

The researcher of [26] propose an optimal power scheduling approach for Demand Response (DR) in a residential community. The article reveals the predetermined power price. The authors of this study formulate the power management issue as an optimization challenge to identify the most favorable schedules. They explore three distinct operating modes, each with its own set of priorities. The first mode prioritizes electricity cost over any discomfort caused, while the second mode prioritizes reducing discomfort above all else. The third option tries to find a compromise between ache and electricity costs. Relying on their proposed planning approach, the outcomes demonstrate a significant exchange between discomfort and energy overhead.

In [27], the problem of scheduling multi-class appliances in multiple residential homes is addressed using MINLP for problem formulation. The authors also presented the PL Generalized Benders algorithm for load scheduling, which addresses the problem in a scattered manner. Each home transmits limited data between the system and the subject in a distributed manner while possessing its private data safe. The proposed method minimizes the cost-to-user comfort tradeoff. The suggested algorithm achieves a near-optimal solution for each habitation according to the results. A DSM model for home users under an RTP pricing model is indicated in [28] to accomplish the three key goals of lowering power bills, PAR, and appliance wait times. GA programming language is utilized to schedule smart appliances, and two scenarios, a single user and twenty users, are simulated to test the effectiveness of the suggested GA-based model.

Introducing a scheduling strategy based on an ideal cessation criterion, [29] classifies users into three clusters based on their power utilization patterns: dynamic, inactive, and moderately dynamic. This user classification process aligns with supply chain segmentation practices, where identifying distinct customer groups is crucial for optimizing resource allocation and demand management.

The authors propose dual scheduling algorithms, employing the altered first come first serve (MFCFS) technique to overcome electricity expenses, which resonates with the supply chain’s focus on cost efficiency. Simultaneously, the early cutoff first (PEEDF) approach is utilized to maximize comfort, reflecting the importance of customer satisfaction and service quality within supply chain operations.

Simulation results exhibit the effectiveness of the suggested algorithms in maximizing comfort while minimizing energy costs, a balancing act similar to supply chain logistics that aim to optimize service levels while controlling operational expenses.

Furthermore, the authors put forward renewable energy and storage models in [30], which align with the growing emphasis on sustainability and green practices in supply chain management. These models present a forecasting technique for electricity load day ahead based on neural networks, showcasing the application of predictive analytics and forecasting within supply chain planning.

The proposed technique anticipates the demand load for the future day using energy consumption patterns from the preceding day and the current day, akin to demand forecasting and inventory management practices in supply chains. Notably, the model not only reduces running time of approaches but also enhances the precision of restricting non-linearity in previous days’ electricity load curve, which is reminiscent of improving forecast accuracy in supply chain demand predictions.

In essence, this research demonstrates how the principles of supply chain management, such as customer segmentation, cost efficiency, sustainability, and demand forecasting, can be applied to the optimization of home electricity management systems.

To balance the residential load and achieve the dual objectives of maximizing electricity cost savings and comfort, GA-based methods for DSM are developed by the authors in [31]. Thermostatically controlled, elastic, and inelastic appliances are divided into five categories. Appliances that are user-friendly and commonly employed. A smart programmable thermostat and a conventional programmable thermostat were created for equipment scheduling. According to simulation findings, the proposed methods lowered cost by 22.63 percent and PAR by 22.77 percent.

The authors of provide the SG power management prototype cite32 for intelligent manufacturing. The activities and resources used in the job are organized based on the TOU’s battery performance electricity price signal. The system model is constructed through the rummage sale of mixed-integer linear programming. For improved computing efficiency, a score status algorithm is proposed. In comparison to the standard heuristic approach, the proposed approach effectively saves more energy while managing extensive assets and task loads [32]. The fetched of savvy domestic stack planning frameworks can be diminished by exchanging the stack to off-peak hours. However, extreme load shifting during these times can result in rebound load peaks, increasing costs. The author provides a prioritized load-carrying power management approach to alleviate these rebounding surges in [33]. To do this, a numerical equation for day-ahead load control is presented. In the enchanting domain of autonomous load shifting, metaheuristic techniques gracefully weave their magic to optimize energy. The proposed method orchestrates a symphony of efficiency, elegantly reducing off-peak load peaks while preserving valuable resources. However, the quest to enlighten power consumers through Demand Response Programs presents a formidable challenge [4]. A visionary model beckons a multitude of participants to embark on a transformative journey, where they can unlock the secrets of electricity prices and the profound impact of their usage. The proposed model differs from the demand flow idea by lowering the maximum power utilization price by cheering users to participate in the DRP [34]. Simulation results support the ability of the recommended model to diminish electricity costs and improve the valley filling of loads.

The triumph of SG hinges primarily on consumer choices. In [35, 36], a compelling proposition emerges, advocating for the integration of energy management in domestic settings to solidify this success. A projected finite machine stands ready to aid in the selection of an optimal scenario tailored to users’ individual preferences. Various approaches have been proposed and tested to assess the continuity and cost-effectiveness of energy use. Simulation results in [37] indicate that the economic scenario reduces electricity costs by 18%. Due to their inherent capacity for self-restoration, self-protection, and self-coordination, metaheuristic models are commonly employed to achieve optimal results in demanding environments. As a result, we used SWA, SSA, and projected SSWA to assess the objective functions of our assignments.

## Problem statement

The four most frequently discussed issues in SG literature are the lowering of PAR, load balancing, consumer ease expansion, and cost minimization. In [7], the integration of RES emerges as a notable challenge within the domain of SG. One approach to address this issue is through the utilization of convex programming, which has the potential to reduce electricity expenses. It is important to keep in that the execution time represents the trade-off because it frequently suffers. Using MILP, ILP, and NILP optimization techniques, [12, 37] tackle the cost reduction problem. On the other side, user convenience and RES integration are disregarded. The study in [10] used MILP to determine the most cost-effective energy consumption pattern while considering integrating renewable energy sources, but without considering user comfort. However, these techniques are limited in their ability to manage multiple appliances. Therefore, ongoing efforts are to develop various meta-heuristic optimization techniques to address this issue. Author in [9] lower the price of electricity using GA. The study’s authors used appliances with the same power rating in various houses. There are appliances in many homes with multiple power ratings. In [14], the author utilizes the MOEA to pursue their objectives of cost optimization and improving user comfort. However, it appears that the consideration of PAR is not explicitly addressed or given significant attention in their approach. Suggest combining DE and HSA methods for the scheduling problem in [17]. The findings imply that this hybrid approach successfully lowers expenses.

In [18], the authors compare their proposed hybrid HSDE technique for day-ahead scheduling with other optimization algorithms combining different optimization methods. These approaches include hybrid artificial intelligence algorithms mixed with contrasting evolution, genital algorithms combined with contrasting evolution, and particle swarm optimizations collaboration with contrasting evolution. The outcome demonstrates that the hybrid HSDE method surpasses these other methods in both cost and computational complexity. In [38–41], the authors utilize ant colony optimization, the GreyWolf approach, and a prior-ity-based energy storage system for scheduling to reduce the PAR and electricity bill. However, these methods do not take into account the typical waiting times.

Subscribers or users face the challenges of balancing electricity costs and user convenience. Many users prioritize cost savings over user comfort, often sacrificing the latter to achieve the former. The current rating system impacts power usage and energy costs, even if shifting the consumption from prime to the off periods may result in electricity cost savings. These factors contribute to the difficulties users face in managing their electricity consumption. According to our research, the proposed HEMS lowers the price of PAR and electricity. We have devised a hybrid method called SSWA to evaluate and optimize the coordination of our HEMS. By incorporating the advantageous features of both SSA and SWA, we aim to achieve superior results in an efficient manner. In our specific circumstances, we take into account several homes. There are various pieces of equipment in every house. Each appliance is broken down into several groups depending on the type of user behaviour. In our research, we extensively considered multiple households and their smart appliances. The project is driven by the following overarching objectives:

- Minimize costs, amplify PAR.
- Harmonize energy distribution through load balancing.
- Discover the perfect harmony between cost and user comfort.
- Choreograph appliance schedules for energy efficiency and waste reduction.

## Problem formulation

The significant objectives are to optimize energy use patterns and reduce PAR and electricity costs. Our optimization problem has been formulated using several knapsack problems (MKP). An MKP is a feasible combinatorial solution that aims to select the most valuable item from a set of options based on their weight and value properties. The primary objective is to keep the net weight of individual items low compared to Knapsack’s weighted capacity by selecting objects with both useful and lightweight attributes. Our optimization challenge aims to lower electricity costs by thoughtfully scheduling appliances for each time slot. Each appliance has two features: a power rating and an on/off status. MKP serves as our guiding light, enabling us to map and address our development problem with precision and optimization:

- The overall quantity of things and devices.
- The item’s and appliance’s weight as well as power rating.
- The cost for each item within each time period.
- The overall amount of time frames and the integer of knapsacks.

Optimization problems are clear as:

$$\begin{array}{c} \text{min}\sum_{tm=1}^{T}M(\sum_{a=1}^{APP}PApp\times sv\left(tm\right)\times ya,tm) \end{array}$$ (1)

Where TM Represents the total number of time intervals or periods considered in the optimization. It could be hours, days, or any other defined time units. AP Denotes the total number of appliances or devices in the home that are being managed by the HEMS. P Indicates the total number of power levels or states that each appliance can operate in. For example, an appliance could have different power levels such as off, low, medium, and high. App Refers to a specific appliance, ranging from 1 to AP, within the home that is being considered in the optimization process. sv(t) Represents the power consumption or energy consumption of the appliance “App” at a particular time interval “t”. This value can be obtained from the appliance’s power consumption profile or measured data. ya, t Represents the decision variable or control parameter that determines the power level or state of the appliance “App” at a particular time interval “t”. It could be a binary variable (0 or 1) indicating the on/off status or a categorical variable representing different power levels/states.

$$\begin{array}{c} Subjectto:L\left(t\right)=E_{in}\left(t\right)+E_{bi}\left(t\right)+E_{b}\left(t\right), \end{array}$$ (2)

L(t) Describes the overall load or energy consumption for the duration of time “t”, Ein(t) denotes the amount of energy used by on-the-go gadgets or appliances over a time period “t”. Ebi(t) Indicates the amount of energy used by constantly on or necessary devices at time interval “t”. Eb(t) Indicates the amount of energy used by background or standby equipment during the time interval “t”. According to the equation, the total load or energy consumption at a specific time period is the sum of the energy used by essential devices (Ebi(t)), background or standby devices (Eb(t), and active appliances (Ein(t)).

$$\begin{array}{c} L\left(t\right)\leq \beta_{th}, \end{array}$$ (3)

L(t): Describes the overall load or energy consumption for the duration of time “t”. It alludes to the volume of energy used or ingested within that specific time frame. Th is the upper limit or threshold for the overall load. The maximum permitted level of energy consumption is established by a fixed figure. According to the Eq 3, the total load at a certain time interval must be lower than or equal to the designated threshold value (th). In other words, the energy usage shouldn’t go beyond the set limit.

$$\begin{array}{c} L_{schtotal}=L_{unstotal} \end{array}$$ (4)

Lschtotal denotes the cumulative energy consumption of activities or devices that have undergone meticulous planning and scheduling. Lunstotal accounts for the overall energy usage attributed to activities or devices that occur spontaneously or lack predetermined plans.

$$\begin{array}{c} C_{schtotal}<C_{unstotal} \end{array}$$ (5)

Cschtotal signifies the overall financial expenditure associated with activities or operations that have been deliberately planned and scheduled. In contrast, Cunstotal reflects the total cost incurred by activities or operations that occur spontaneously or lack a predetermined plan. The equation emphasizes that the accumulated cost of scheduled activities is lower than the cumulative cost of unscheduled activities. This comparison sheds light on the financial benefits of careful planning and scheduling. By understanding the relationship between scheduled and unscheduled costs, we can optimize financial management and identify opportunities to minimize expenses. It underscores the significance of effective planning in achieving cost efficiency.

$$\begin{array}{c} t\alpha <t<t\beta \end{array}$$ (6)

The expression specifies the temporal range within which the time variable (t) exists, bounded by the minimum value t*α* and the maximum value t*β*. It signifies that the value of t lies within this interval, excluding the extreme values of t*α* and t*β*. It indicates that the time variable (t) falls between t*α* and t*β*, In this captivating mathematical equation, *t*_*β*_ and *t*_*β*_ converge, casting a spotlight on a precise timeframe, wherein the time variable unfolds its graceful symphony. This equation is particularly useful for setting temporal boundaries or limitations when analyzing data or performing calculations within a specific time range. It enables us to establish the temporal scope or constraints for the time variable being considered.

$$\begin{array}{c} sv(t)\in [0,1] \end{array}$$ (7)

The importance lies in the decision variables Sv(t) and ya,t within the domain of scheduling devices and distributing energy. The state or condition of each device at a specific moment is denoted by Sv(t), while ya,t represents the allocated energy for individual devices during that time interval. It is through these decision variables that the overall energy management process is optimized. The optimization approach is centered around the selection of suitable values for Sv(t) and ya,t, aiming to achieve an energy-efficient system. By making well-informed decisions based on these variables, the determination of when and how much energy should be allocated to each device is effectively carried out by the system. As a result, the overall energy consumption and efficiency of the system are significantly influenced. Sv(t) and ya,t serve as crucial factors enabling the system to make informed choices regarding device scheduling and energy distribution. This, in turn, leads to improved outcomes in energy management.

The goal function denoted by Eq (2), which determines the total amount of energy used by all appliances over a time period of t minutes, is used to minimize the daily cost. The objective function’s constraints are defined by Eqs (2) and (7). Over time, Eq (2) expresses the overall consumed energy of in-circuit appliances. In order to lower PAR, Eq (3) limits the amount of energy consumed during a specific time period to a predetermined level. The control utilization restriction in Eq (4) makes it beyond doubt that the control utilize does not alter due to planning or the length of time the device is used.

According to Eq (5), the cost of a scheduled weight each day should be less than the price of an unforeseen weight each day. Within the realm of equations, behold the enchanting Eq (6), where the symbol t unveils the very inception of an appliance, while its counterpart t gracefully heralds its final curtain call. As we delve deeper into this mathematical tapestry, Eq (7) takes center stage, illuminating the current state of the appliance. With an exquisite binary portrayal, it unveils a world where the appliance may gracefully assume the positions of 0 or 1, casting an intriguing spell upon our mathematical modelling. If the value is 1, the appliance is on; if it is 0, it is off.

## System model

**Fig 1:**
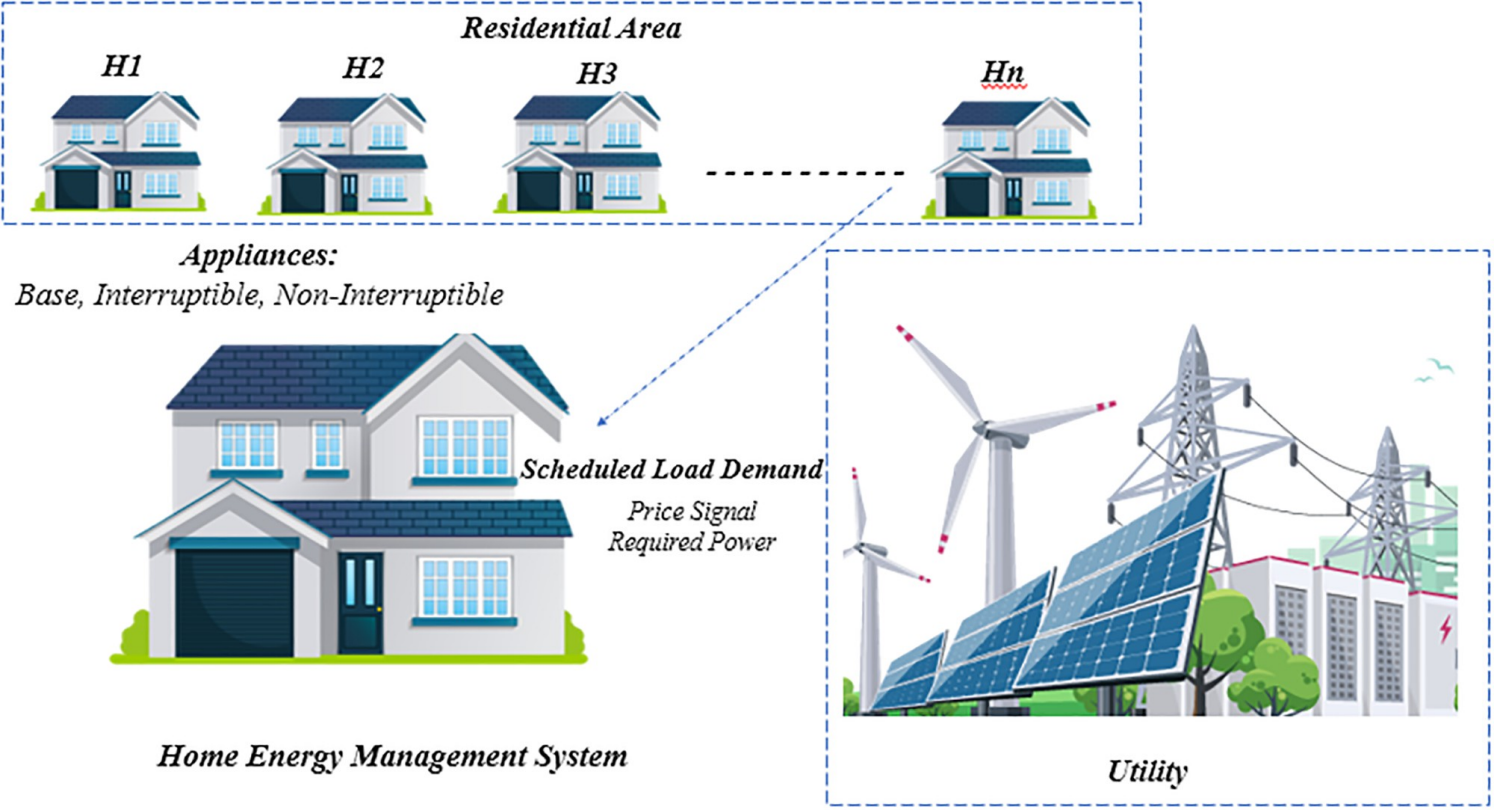
Proposed HEM system model.

A thorough explanation discusses in this section of the proposed work. Fig 1 illustrates that many residential areas receive utility tariffs, and we propose a HEMS to plan domestic applications, ultimately reducing PAR and electricity bills. The system comprises EMC, SM, and smart appliances within a smart home. SM facilitates both the electricity supplier and the customer, while EMC schedules equipment according to the pricing indication provided by the electric company. EMC, the master conductor of energy management, receives consumption trend information of equipment. Aligned with the utility’s signal price, EMC schedules operations accordingly. The utility sends price information to SM, which in turn transmits it to EMC. It simultaneously gathers information from EMC about energy use and transmits it to the utility. In the expansive vast of connectivity, traditional protocols and wireless networks like Z-wave and ZigBee establish bridges between the orchestrators (e.g., EMC), intermediaries (e.g., Smart Module), and the essential devices that power our homes. Within this intricate network, a dynamic ecosystem enables fluid data exchange, allowing the orchestrators, intermediaries, and devices to communicate, synchronize, and harmonize seamlessly. The study considers several homes, each containing 12 appliances. RTP tariffs are used to calculate electricity bills. In our example, a choice may be taken every 12 minutes, and the scheduling horizon is 12 minutes rather than one hour because only a few appliances operate for not more than one hour. Separating a whole day into 120 equal time slots, each lasting 12 minutes, enables us to reduce electricity costs while improving system reliability. We employ the RTP pricing mechanism, which is the most successful in the power market. RTP pricing signals are subject to hourly fluctuations. In an RTP pricing scheme, price signals ebb and flow, varying across different time slots. However, once within a particular time slot, these price signals remain steadfast and unwavering, providing a constant reference for energy pricing during that specific period.

In the suggested system, users possess single or multiple residences, each adorned with a diverse array of appliances that can be categorized into three distinct groups based on their usage patterns and characteristics: foundational appliances, steadfast appliances, and flexible appliances. These intelligent devices are thoughtfully segregated into multiple subgroups, taking into account their power consumption patterns for the purpose of efficient scheduling. Our model aims to alleviate PAR concerns and minimize electricity expenses, while also prioritizing UC by considering waiting times. To gain a comprehensive understanding, please refer to Table 1, which presents a comprehensive list of appliances, power assessments, and their corresponding operating times (LOT). For evaluation, we implemented three scenarios in a smart home with multiple appliances using SSA, SWA, and hybrid SSA and SWA along with RTP tariff: For 12 appliances, the findings are determined by expense, PAR, energy usage, and user comfort.

**Table 1:** Appliance parameters.

Appliance	Time Flexibility (hours/slots)	Power Consumption (kWh)	Operational Classification
Humidifier	0.05, 0.05, 0.08	25	Adjustable/Adaptable
Dishwasher	0.6, 1.2, 1.4	15	Adjustable/Adaptable
Electric radiator	1.8, 2.5, 2	16	Adjustable/Adaptable
Electric Kettle	1.5, 1.2, 1.4	19	Adjustable/Adaptable
Water pump	1.7, 2.2, 4	35	Adjustable/Adaptable
Water Cooler	0.05, 0.08, 0.1	25	Adjustable/Adaptable
Washing Machine	0.4, 0.5, 0.7	6	Non-Interruptible
Cloth dryer	0.9, 1.0, 1.2	6	Non-Interruptible
Air conditioner	1.5, 1, 4, 1	25	Adjustable/Adaptable
Light	0.3, 0.4, 0.5	65	Base
Water heater	1.5, 2, 4.5	16	Adjustable/Adaptable
Refrigerator	0.2, 0.3, 0.4	125	Base

### Load classification

Apparatuses are categorized into three types established on power consumption patterns: Basic device, variable load device, and the static load device.

#### Interruptible appliances

There are several parallels between interruptible and deferrable appliances. Heavy loads require more than 1.5 kWh of power to operate continuously. To meet the objectives of reducing costs and PAR, Performing two big loads simultaneously is not recommended. This study defined heavy loads as electric radiators, water heaters, and water pumps. To maintain efficient energy management and prevent potential conflicts or overloading, it is recommended to deactivate the water heater when using an electric radiator and water pump simultaneously. Interruptible appliances are denoted by ‘IN’, and their power usage is represented by ‘Ein’. The following formula is used to determine the daily energy consumption of any interruptible device with a energy rating of “in”:

$$\begin{array}{c} \;\text{Ein}\;=Tm\;\sum tm=1\;\sum \;\text{in}\;\epsilon \;\text{IN}\;\rho \;\text{in}\;\times \text{svin}(t) \end{array}$$ (8)

Within the context of our system, the variable Tm represents full-time slots. Svin(tm) denotes the assumption of the existence of an individually interruptible appliance during a specific time slot t. The variable sv in(tm) takes binary values, where 0 signifies that the appliance is off and 1 indicates that it is turned on. By employing these variables, we can effectively monitor the status of the interruptible appliance throughout the designated time slots.

#### Non-interruptible appliances

Once started, these appliances cannot be stopped and relocated to another time period. Examples of such devices include washing machines and clothes dryers. Non-interruptible appliances are denoted by ‘NI’, and their energy consumption is represented as ‘Eni’. The total power usage of each non-interruptible application with a power rating of ‘ni’ is calculated using the following formula:

$$\begin{array}{c} \;\text{Eni}\;=T\;\sum t=1\;\sum \;\text{ni}\;\epsilon \;\text{NI}\;\rho \;\text{ni}\;\times \text{svni}(t) \end{array}$$ (9)

An interruptible appliance’s condition at a specific time ‘t’ is represented as svni(t), and may be expressed as follows

$$\begin{array}{c} svni(t)=appliancesstate0representsoffand1representsON. \end{array}$$ (10)

#### Base appliances

It cannot be managed and is frequently referred to as a fixed type of equipment. It is impossible to alter this equipment’s energy consumption patterns or overall working time. Some appliances must be switched on before a user can turn them on. A lightbulb, an air circulator, a TV, and a fridge serve as some instances. “B” represents fundamental devices, while “Ec” symbolizes their energy consumption. The energy usage of the standalone fundamental device bA is denoted as ‘a’. The subsequent equation is employed to calculate the aggregate energy consumption:

$$\begin{array}{c} \text{Ec}=\text{N}\sum \text{n}=1\sum \text{a}\in \text{A}\eta \text{a}\times \text{sva}(\text{t}) \end{array}$$ (11)

In the equation, Tm represents the total number of time slots. The variable sva(n) indicates the state of an individually interruptible or suspended appliance during the ‘n’ time slot. It is assumed as follows:

$$\begin{array}{c} svb(t)=appliancesstate0representsoffand1representsON. \end{array}$$ (12)

## Optimization approaches

Because of their predictable nature, conventional optimization techniques like MILP, ILP, and MINLP are computationally inefficient when it comes to real-time optimization. These traditional methods encounter difficulties in effectively managing a substantial quantity of appliances. As a result, we are utilizing the SSA and SWA metaheuristic methodologies to achieve our objectives. The outcomes of a blend of SSA and SWA are contrasted with those of typical SSA and SWA in order to assess User satisfaction, PAR, and Expense. The subsequent subcategories elaborate on the chosen meta-heuristic algorithms.

### Social spider algorithm

The Social Spider Algorithm (SSA) is an optimization algorithm miming social spiders’ hunting and foraging behaviour. The approach generates a population of spiders in the solution space with random placements. The spiders follow a set of movement rules to move around and hunt for prey in the seek arena. The evaluate function is used to assess each spider’s fitness. In each iteration, the spiders search for prey using their movement rules. If a spider’s new position falls outside the search space, it is returned in. The spider’s position and strength are updated if the novel suitability circumstance is higher existing suitability circumstance. After all of the spiders have relocated and upgraded their positions, they are organized by decreasing rank by fitness. The new position relies on the existing position of each spider and is calculated using Eq (14).

$$\begin{array}{c} y_{j}\left(t+1\right)=y_{j}\left(t\right)+u_{j}\left(t+1\right) \end{array}$$ (13)

Current position refers as *y*_*i*_(*t*) of spider i at time t, *v*_*i*_(*t* + 1) indicate the velocity of spider i at time t+1, and at time t+1, *y*_*i*_(*t* + 1) denotes new position of spider i.

The higher-ranked spiders influence the motion of the lower-ranked spiders. Each spider’s position is updated based on the movements of the higher-ranking spiders. If a spider’s new position takes it outside the search space, it is wrapped back in. If the new position’s fitness is preferable to the spider’s actual state, the spider’s position and fitness are revised.

The pheromone trail left by each spider is determined using Eq (15) based on its fitness level:

$$\begin{array}{c} b_{-}i\left(t+1\right)=\left(1-a\right)*b_{-}i\left(t\right)+a^{*}\text{delta}\;b_{-}ij \end{array}$$ (14)

Where the spider left the pheromone at j position at t time is *b*_*i*_*j*(*t*), the pheromone amount of pheromone spider I that is left at position j, based on its fitness and the fitness of the subsequent spiders, is represented by delta b ij and the evaporation rate is given by rho. The algorithm runs for the stipulated number of iterations before returning the spider with the fittest as the solution.

**Algorithm 1** SSA for SG scheduling

Begin by initializing spider populations and pheromone trails.

Determine the number of spiders, denoted as N.

Randomly set the initial positions of spiders, denoted as x_i, in the search space.

Initialize pheromone paths, denoted as tau_ij, with a low value.

Assess each spider’s fitness by using the evaluation function.

Repeat the following steps until the stopping criteria have been met:

Update the velocity of every spider using the movement equation:

*v*_*i*_(*t* + 1) = *sum*_*k*_ = 1^*N*^(*A*_*ij*_(*t*) * *delta*_*x*_*ij*_(*t*)/*D*_*ij*_(*t*)) + *rand*()*r*(*x*_*best* − *x*_*i*_(*t*))

where A_*ij*_(t) represents the pheromone intensity between spiders I and j at time t, delta *x*_*ij*_(t) represents the difference between spider I and j’s positions at time t, *D*_*ij*_(t) indicates the gap between spiders I and j at time t, r is a criterion, and x best represents the best position found thus far.

Update the location of each spider using the velocity:

x_i(t+1) = x_i(t) + v_i(t+1)

Assess each spider’s fitness by using the evaluation function.

Update the pheromone trails using the pheromone updating equation:

*tau*_*ij*_(t+1) = (1-rho)*tau*_*ij*_(t) + rhodelta_*tau*_*ij*_

Update the best position discovered thus far:

If a spider finds a better alternative than the current best, update x_best.

Return the optimal solution discovered.

End.

### Strawberry Algorithm (SWA)

The Strawberry Algorithm is a metaheuristic optimization algorithm that takes inspiration from the foraging behaviour of strawberry plants. To begin the procedure, a collection of individuals with locations within the search space is formed at random. During each cycle, each individual selects a neighbour at random and produces a new position depending on the location of that neighbour. If a novel location fit is superior to the person’s existing location fit, the fit and location are updated. The people are then ranked in downward order depending on their fitness. The best individual is then employed to direct the movement of the rest. Each person creates a new position based on the top person’s work and the distance between them. If the new location fit is higher than the person’s fit, the person’s location and fit are updated.

Apply Eq 16 to determine the novel location of each plant within the examination area and formulate it:

$$\begin{array}{c} x_{i}\left(t+1\right)=x_{i}\left(t\right)+v_{i}\left(t+1\right) \end{array}$$ (15)

A new velocity for each asset is calculated using Eq 17, based on current velocity, individual best site, and global best site. It is formulated as

$$\begin{array}{c} v_{i}\left(t+1\right)=wv_{i}\left(t\right)+c1r1*\left(p_{i}\left(t\right)-x_{i}\left(t\right)\right)+c2r2\left(g_{b}est-x_{i}\left(t\right)\right) \end{array}$$ (16)

Cognitive and social learning variables are c1 and c2, respectively, whereas random values between 0 and 1 are used for r1 and r2. The inertia weight is W. The variables x i(t), p i(t), and g in this Equation accurately depict the current location at time t, the plant i’s individual best position at time t, and the best position on Earth at this moment. The new plant position, x_i(t+1), is calculated using the outcome velocity, v_i(t+1).

The site of a plant in the examination domain determines its appropriateness value. The fitness value is determined by the optimization issue at hand, which is problem-specific and fluctuates. Considering each plant’s current position and fitness value, the above Equation determines its personal best position.

$$\begin{array}{c} if\left(fitness\left(x_{i}\left(t\right)\right)>fitness\left(pbest_{i}\right)\right)thenpbest_{i}=x_{i}\left(t\right) \end{array}$$ (17)

The fitness value of agent i in its current position is denoted by fitness(x_i(t)). The ideal global position updated created on the personal best positions of all plants, using the following Equation:

if (fitness(pbest i) greater than fitness(gbest)) then gbest = pbest i, (19) Fitness(pbest) Fitness(gbest) represents the fitness score of the world’s greatest population placement of plants, and I represent the fitness score of the individual best placement of plants. Provides a graphical representation of the process.

**Algorithm 2** SWA for SG scheduling

Make the initial plant populace:

For each plant I in the range 1 to N:

Set x i randomly within the position range.

Set v i randomly within the range [-pos range, pos range].

Establish a personal best position and fitness level for each plant:

Set pbest i to x i for each plant I.

Set fitness pbest i equal to the appropriateness of x i.

Acquire best appropriateness and site in the world:

Set fitness gbest to the fitness of x 1 and set gbest to x 1.

For each plant I in the range 2 to N:

If fitness(x i) is greater than fitness gbest, then set gbest to x i and fit gbest to fit(x i).

Enter the main loop:

For each time step t in the range 1 to max iteration:

For each plant I, do the following:

Calculate the novel velocity “v I” using the velocity equation.

Calculate the new position x I utilizing the position condition.

Calculate the wellness worth of the new position, fitness(x i(t+1)).

If fitness(x i(t+1)) is greater than fitness pbest i, the individual best position and fitness are upgraded.

If wellness pbest I is greater than wellness gbest, update the worldwide best position and wellness.

Display the ongoing worldwide best position and wellness.

Provide the ultimate position and fitness ranking in the world.

End

### Hybrid SSA and SWA

The hybrid approach combines two methods in which, the SSA and SWA operation. This section thoroughly examines the suggested amalgamated approach. It is possible to improve search performance by combining the unique characteristics of the SSA and the SWA. Spiders and strawberry populations coexist and move around in the search space in this hybrid algorithm. We adjust each spider’s position according to its movement guidelines and each strawberry’s position according to its neighbour in each iteration. The spider and strawberry populations are then sorted according to fitness ratings, and the higher-ranked individuals are used to direct the movement of the lower-ranked ones. The hybrid algorithm combines the strawberry neighbour-based movement for local exploitation with the spider movement principles for global exploration. This combination creates a search area where exploration and exploitation can coexist to enhance convergence to the best outcome. Algorithm 3 explains the crossover approach.

**Algorithm 3** SSWA for SG scheduling

Begin

Arbitrarily initialize the position of each insect within the desired run.

Randomly set the velocity of each spider I within the range of [-pos range, pos range].

Assign a random value between [0, 1] to each spider’s social skill b i.

Set the initial values for fitness and personal best position for each spider I.

Set x i = pbest i for each spider I.

Assign Fitness(x i) = fitness pbest i for each spider I.

Determine the global best position and fitness level.

Set Fitness gbest = fitness(x 1) and gbest = x 1.

For each spider I between 2 and N:

Update gbest = x i if fitness(x i) greater than fitness gbest.

Set Fitness gbest equal to Fitness(x i).

Compute the new position for each spider using the SSA position equation for each iteration t between 1 and max iter.

Update the social ability b i for each spider I using the SSA social ability update equation.

Evaluate the appropriateness value to the novel site using the problem-specific fitness function.

Update the personal best position if the fitness value of the new position fitness(x i(t+1)) is higher than the personal best fitness value fitness pbest i.

If the personal best fitness value fitness pbest I is higher than the global best fitness value fitness gbest, update the global best position.

Calculate the new velocity v i for each spider I using the SWA velocity equation.

Compute the appropriateness value for new site fitness(x i(t+1)).

Change and update the personal best position if the new position’s fitness value, fit-ness(x i(t+1)), exceeds the previous position’s fitness value, fitness pbest i..

Update the worldwide best position if the individual’s best wellness pbest I is more noteworthy than the worldwide best wellness gbest.

Update the position of each spider I using the SWA position equation.

Display the current position and fitness level.

Output the final, fittest position.

End

**Fig 2:**
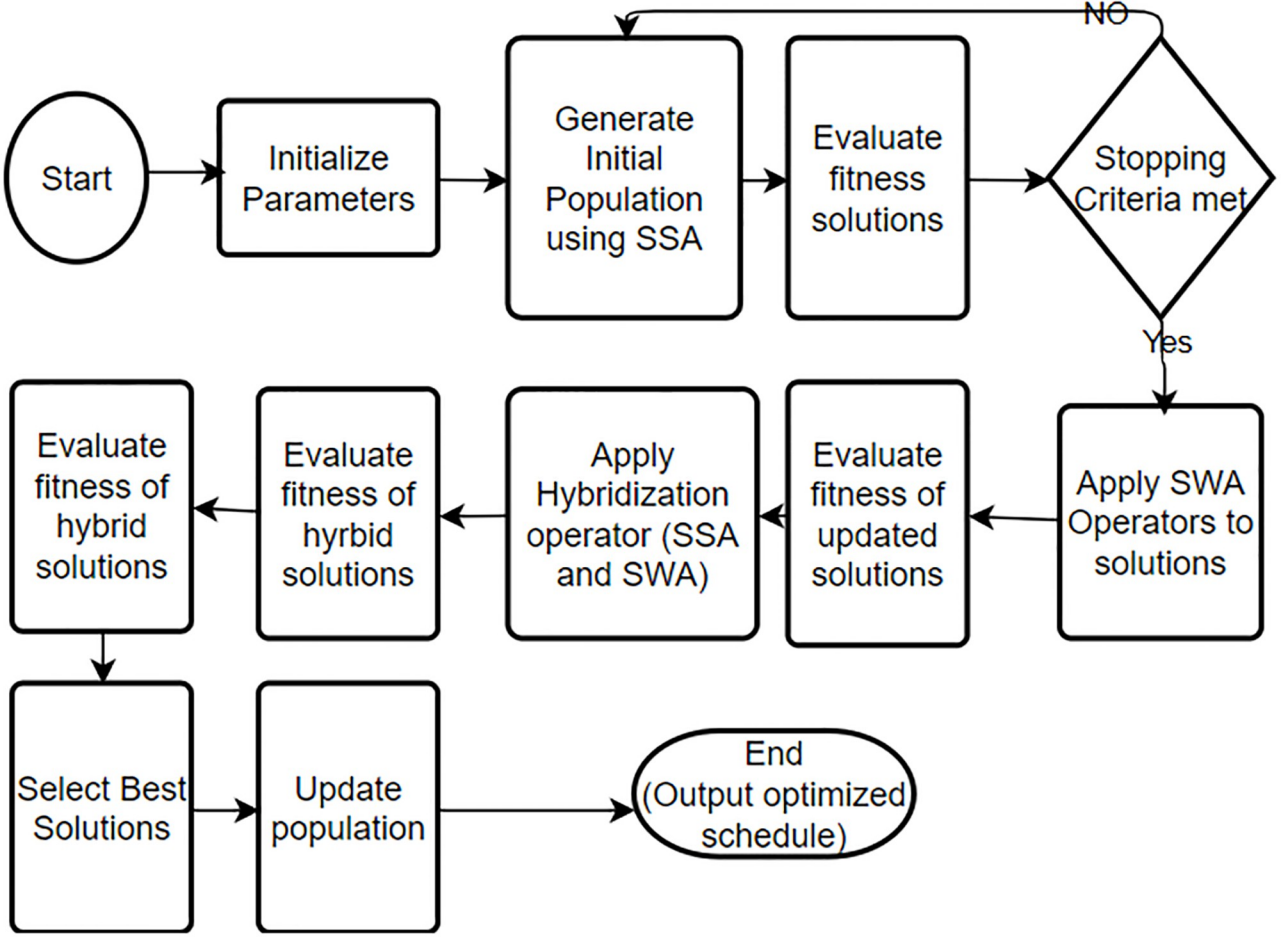
Proposed HEM optimization flowchart.

The presented HEC optimization flowchart is depicted in Fig 2. The recommended approach proves to be economical as it reduces both PAR and power expenses, while also taking into account user satisfaction and waiting duration. The proposed SSWA approach is further explained in Algorithm 3. The hybrid method is assessed based on PAR, load, power cost, waiting time, and user comfort outcomes. Multiple scenarios are taken into consideration to simulate and evaluate the results of SSA, SWA, and their amalgamation. Appliances are scheduled in different time slots to optimize energy consumption and save money on electricity. Table 1 outlines the characteristics of each equipment used to determine electricity costs, including the appliance status and power rating. The electricity cost is computed using the Eq (18), whereas PAR and Load are determined using Eqs (20) and (19), respectively.

$$\begin{array}{c} \;\text{Cost}\;=\sum_{t=1}^{24}E_{\text{Rate}\;}^{\text{hour}\;}*P_{\text{Rate}\;}^{App} \end{array}$$ (18)

$$\begin{array}{c} \text{Load}=P_{\text{Rate}\;}^{\text{App}\;}*App \end{array}$$ (19)

$$\begin{array}{c} \text{PAR}=\frac{\text{max}({\text{Load}}_{S})}{\text{Ave}({\text{Load}}_{S})} \end{array}$$ (20)

#### Encoding strategy

In our proposed SSWA (Social Spider and Strawberry Algorithm) for home energy management, we utilize a binary representation as the encoding strategy. This means that each chromosome or solution in the optimization process is represented as a string of binary digits. In the binary encoding strategy, each bit in the chromosome corresponds to a specific decision variable or parameter in the home energy management problem. The value of each bit represents the on/off state of a particular device or the allocation of energy to that device during a specific time interval. For example, if we have n devices to be scheduled and m time intervals, the chromosome length would be n * m, with each bit indicating the state or energy allocation for a specific device at a specific time interval. A “0” could represent the device being off or no energy allocated, while a “1” could represent the device being on or energy being allocated.

The binary encoding strategy allows for efficient manipulation and evaluation of solutions during the optimization process. It enables the algorithm to explore different combinations of device states and energy allocations, seeking the most optimal solution that minimizes energy consumption or achieves other defined objectives. By utilizing the binary encoding strategy in SSWA, we can effectively model and solve the home energy management problem, finding efficient schedules and energy allocations that result in reduced energy consumption and cost savings.

## Simulation and results

This section contains a comprehensive examination of the simulation results. Our evaluation is based on MATLAB simulations, which assess the effectiveness of the proposed SSWA strategy and the SSA and SWA algorithms. Taking into account electricity costs, energy consumption, PAR, and user comfort, the assessment involves simulations for households of different sizes. Each household is equipped with a diverse range of appliances, each with unique power ratings. Table 1 provides a comprehensive overview of appliance types, their corresponding power levels, and Length of Time (LoT). Subsequent sections delve into the simulation results obtained to achieve our desired objectives.

### Energy consumption

**Fig 3:**
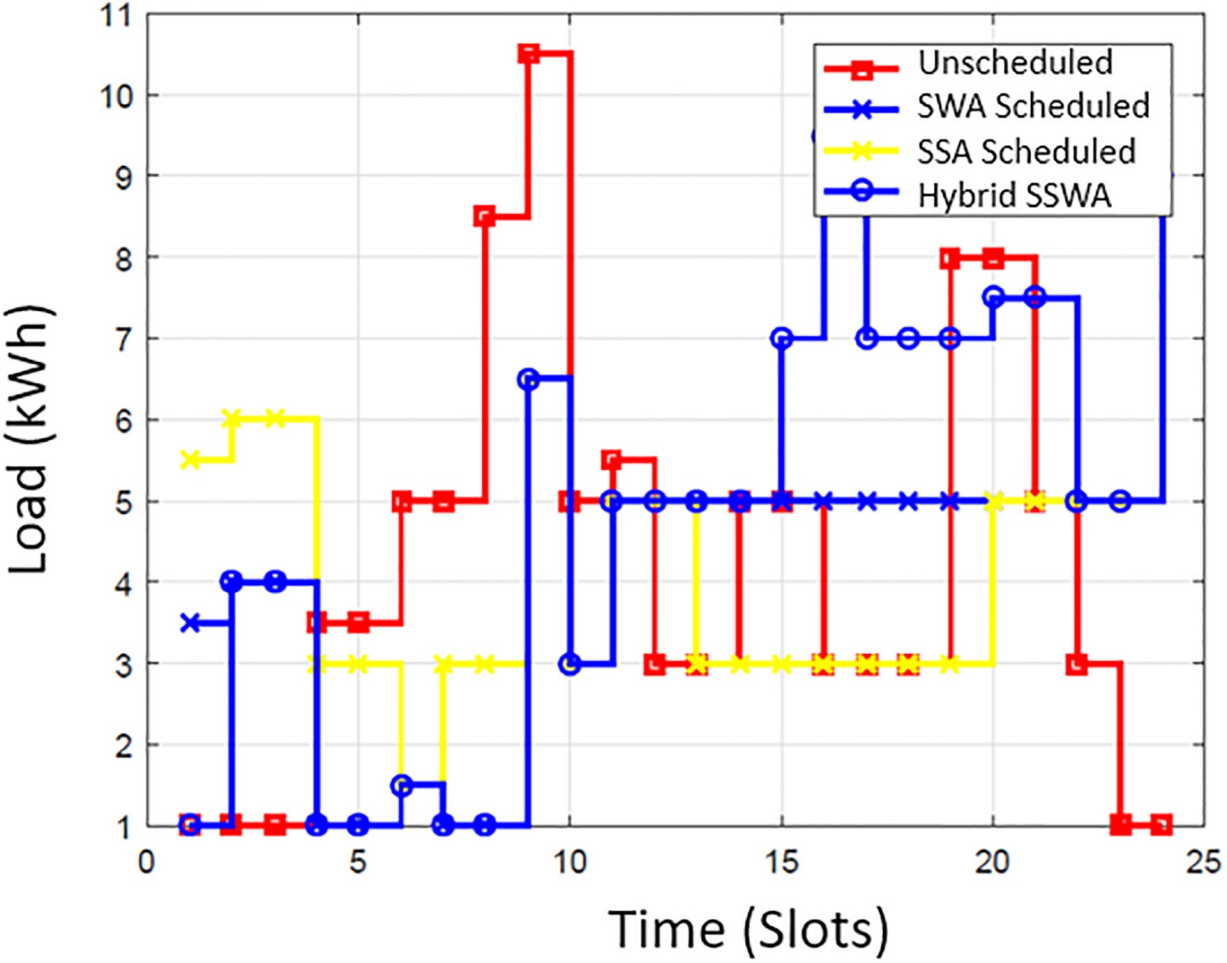
Single house power consumption.

**Fig 4:**
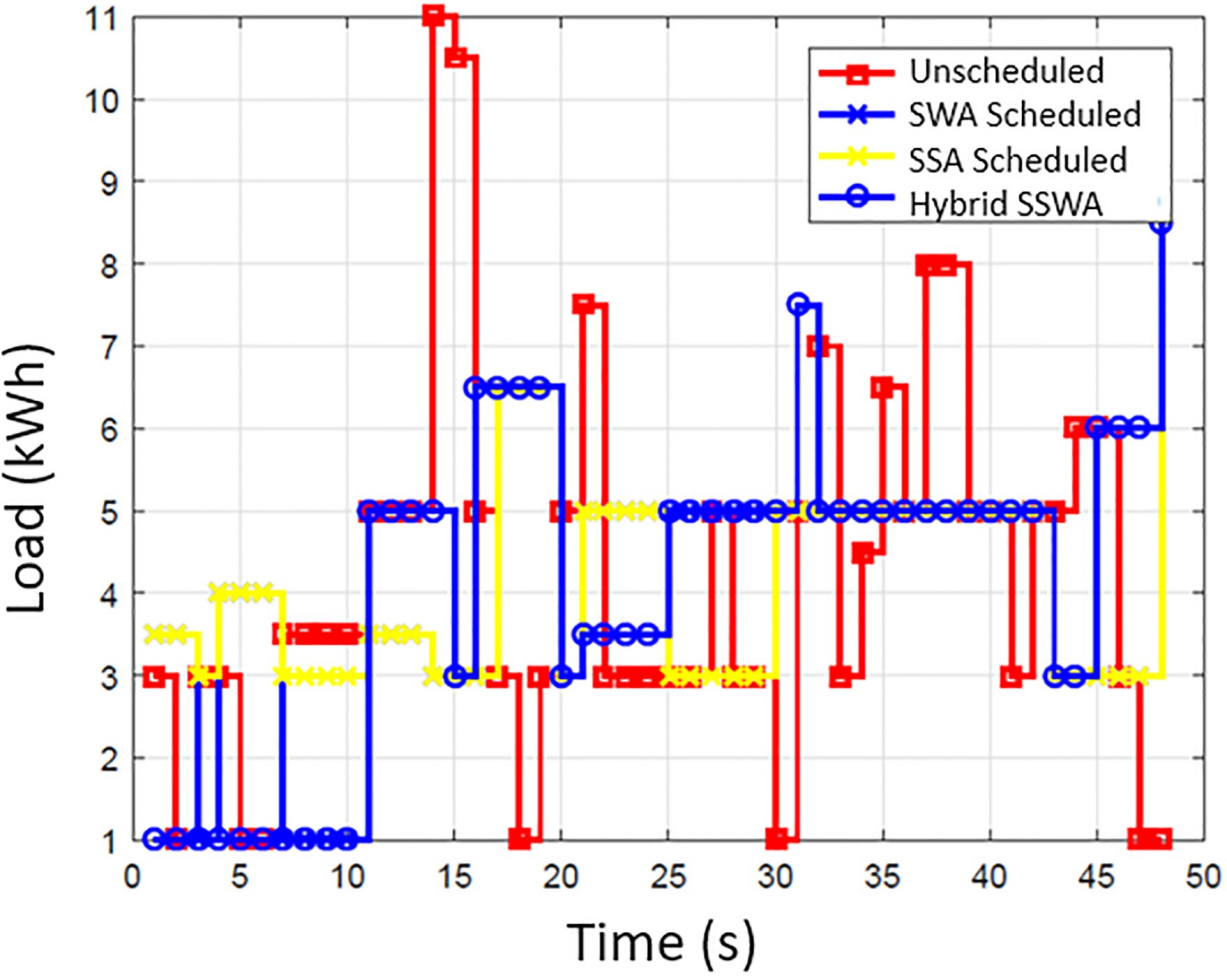
10 houses power consumption.

**Fig 5:**
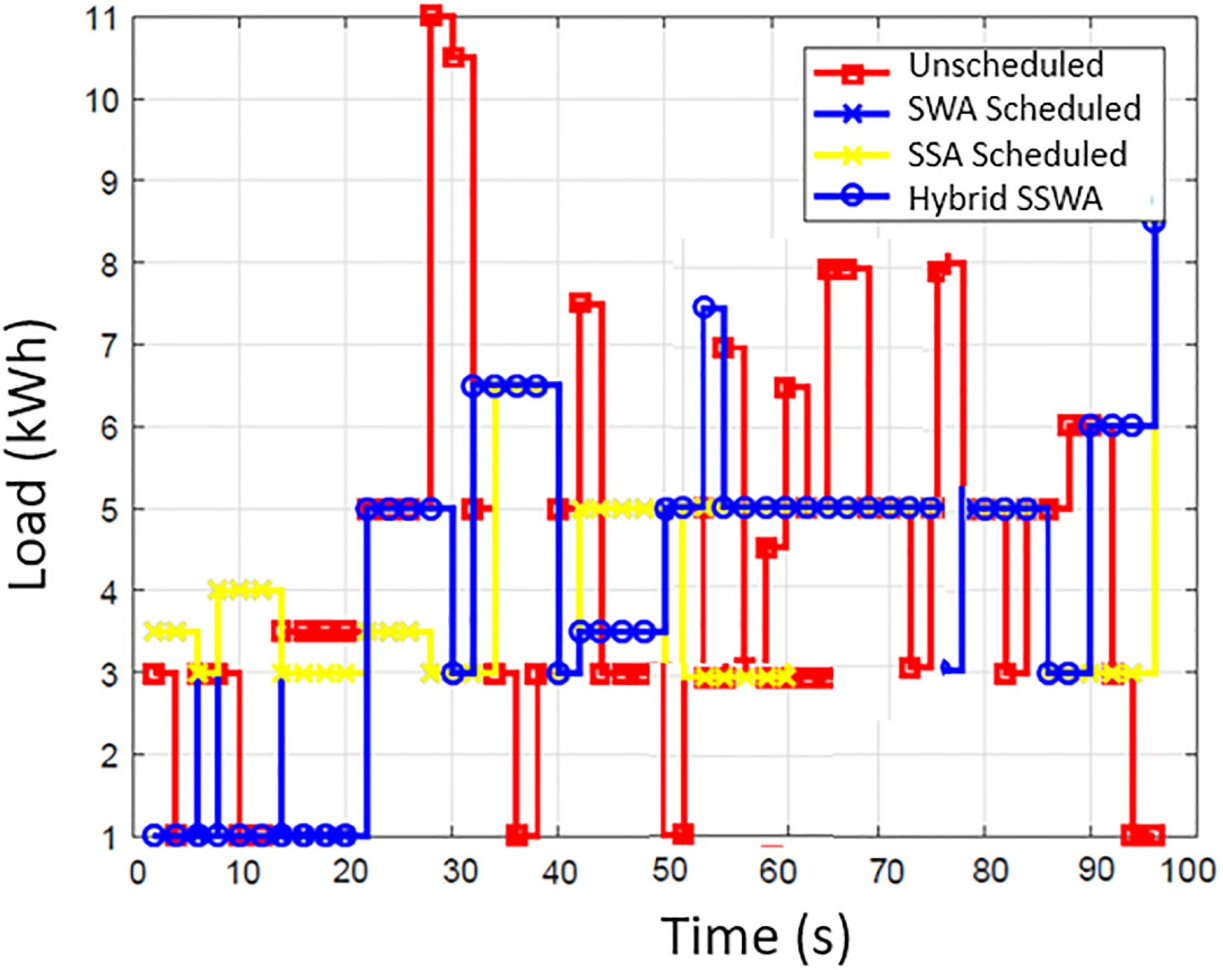
50 houses power consumption.

**Fig 6:**
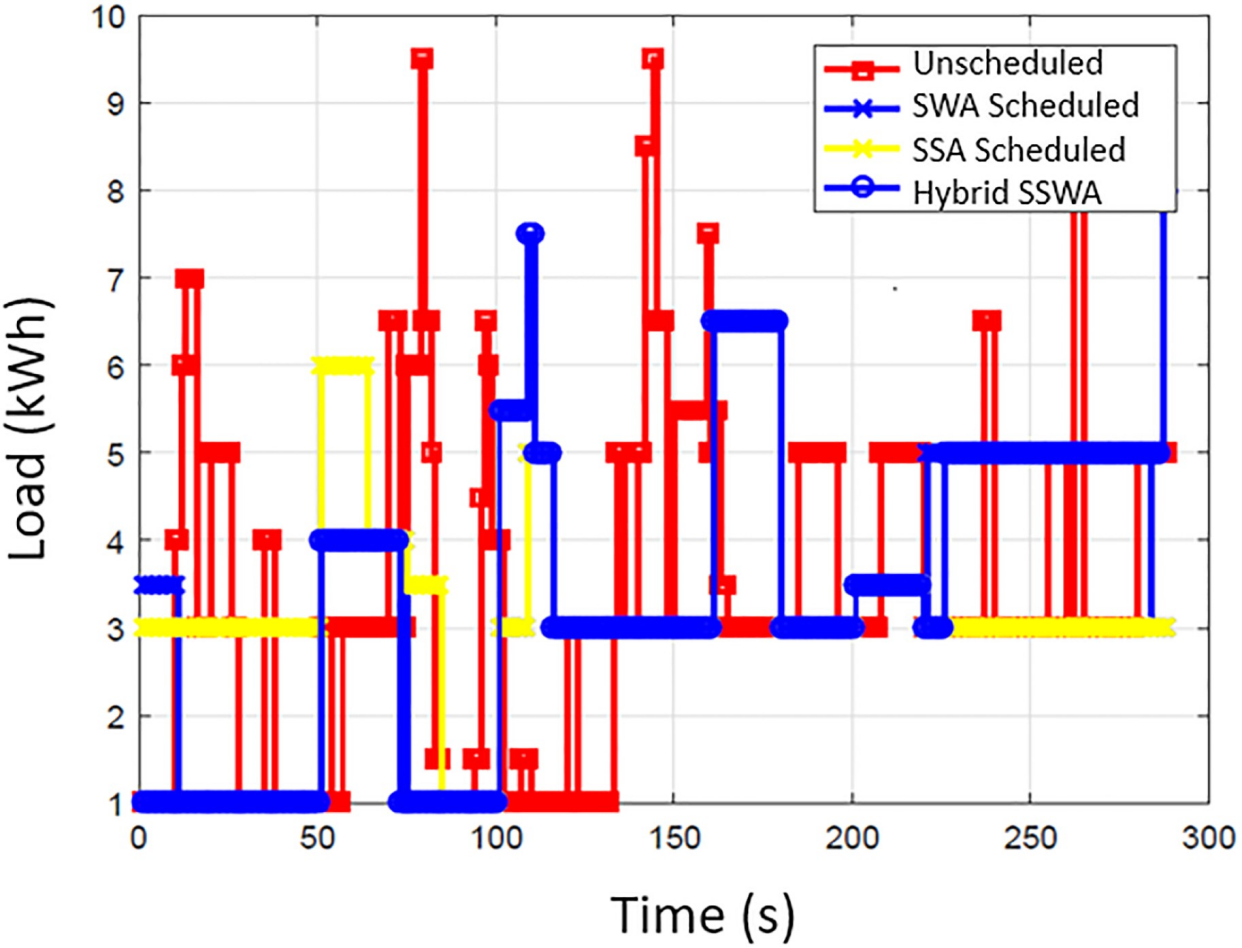
100 houses power consumption.

The energy consumption pattern before and after the scheduling for each time slot is illustrated in Fig 2a. The simulation outcomes show that the suggested SSWA technique effectively achieves the necessary load scheduling goals. By strategically avoiding peak energy consumption during specific times of the day, SSWA successfully reduces the total number of peaks compared to other techniques. Additionally, the load consumption pattern achieved through the implementation of scheduling algorithms demonstrates significant improvement over the unplanned load pattern. Effectively moving the majority of the load from a high point to go off hours results in a regular energy consumption pattern that is more efficient. While load shifting lessens consumer comfort, it also results in cost savings. Consumer will save more money by shifting their load and being flexible with their energy usage plan. The power consumption trends of ten, fifty, and one hundred households are depicted in Figs 3–6, respectively. Regarding power use patterns, our proposed method performed better than the others indicated.

### Cost

**Fig 7:**
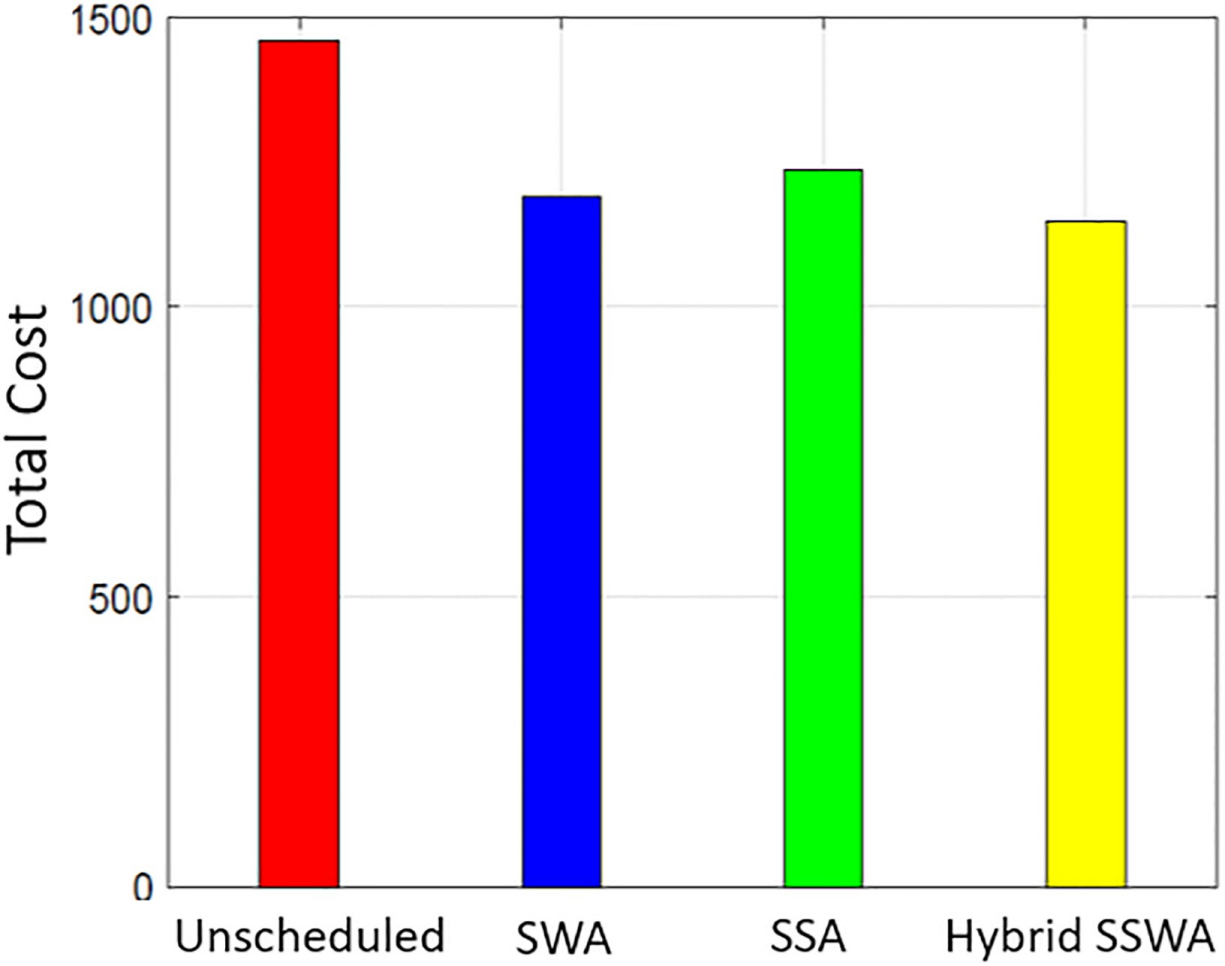
Single house total cost.

**Fig 8:**
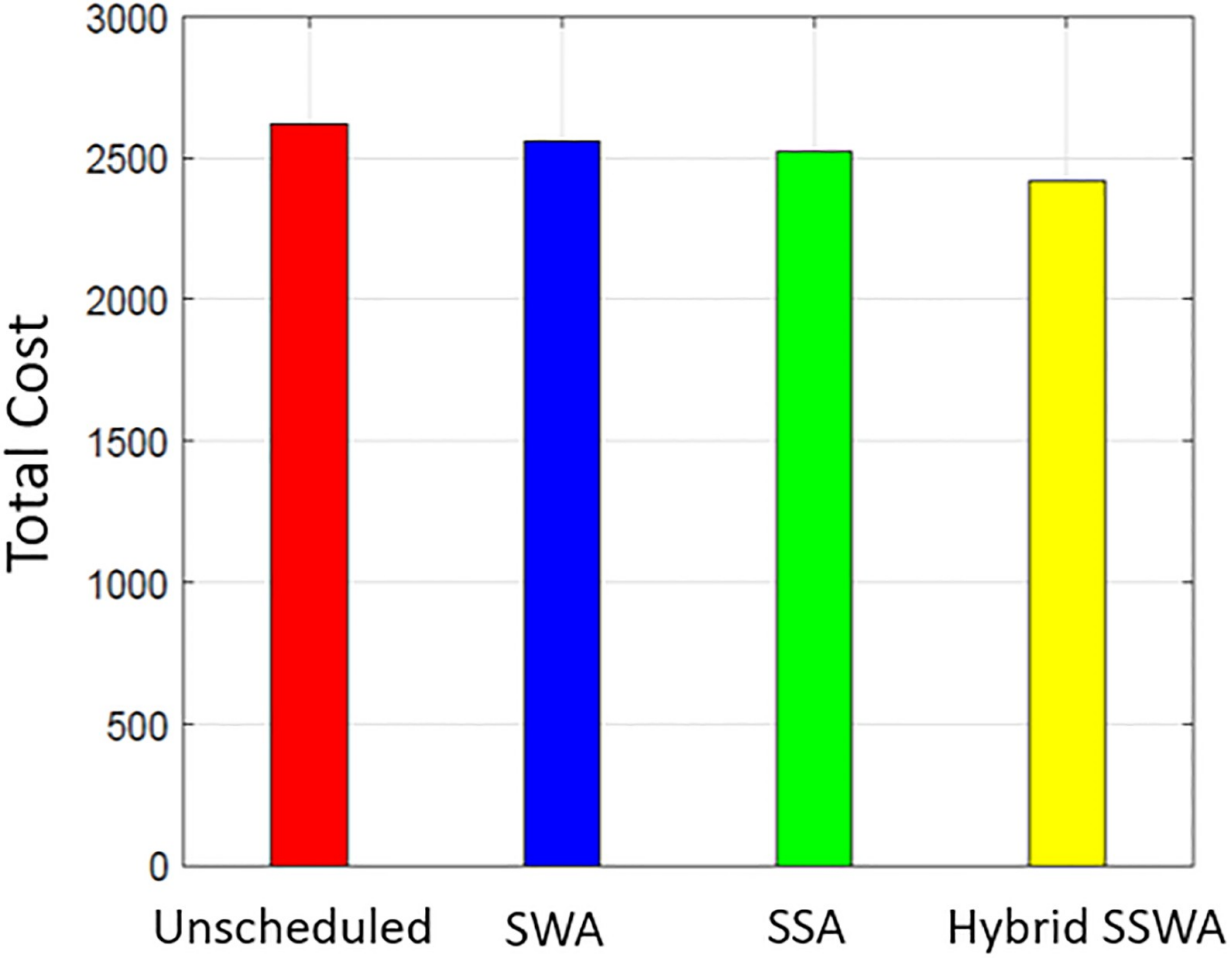
10 house total cost.

**Fig 9:**
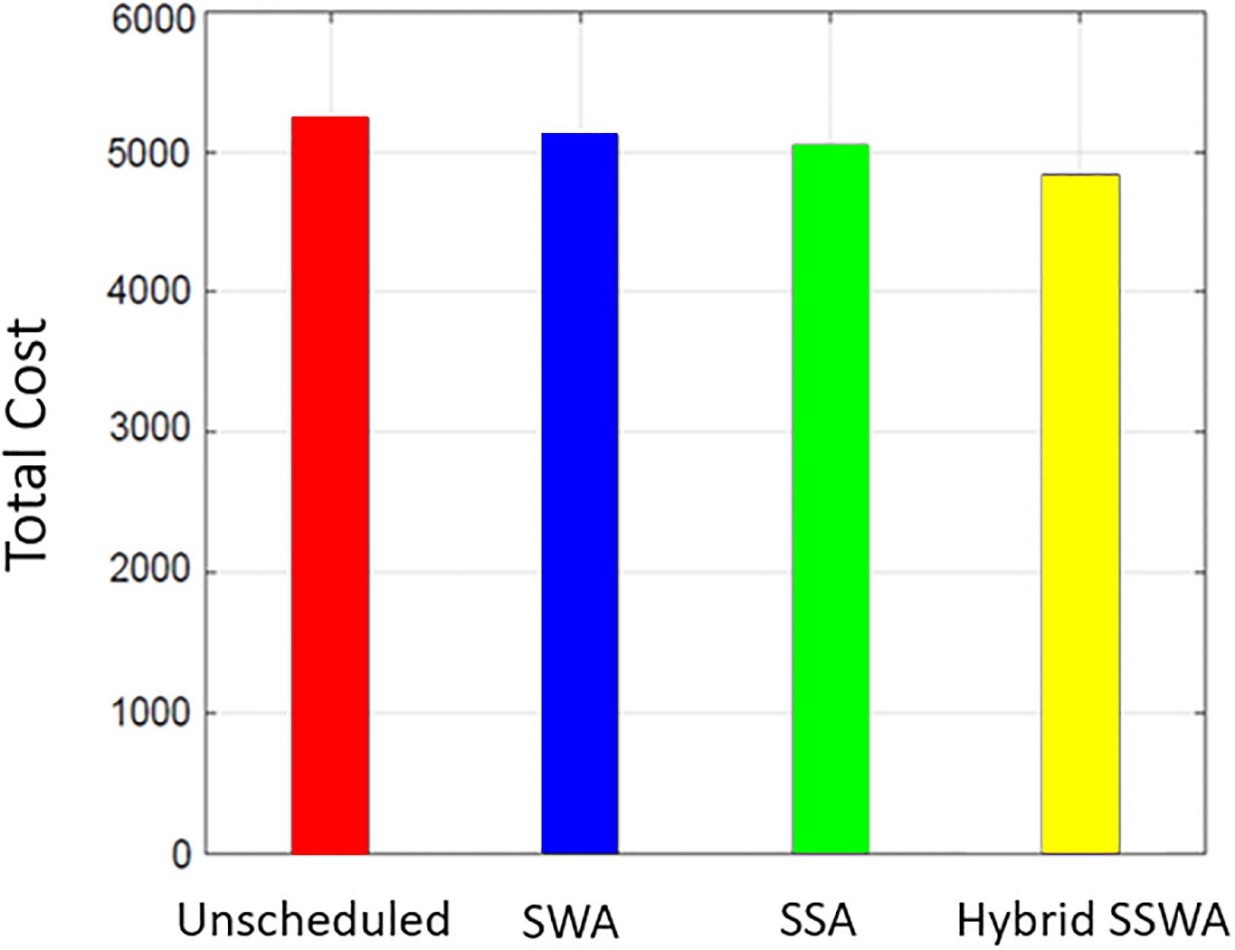
50 house total cost.

**Fig 10:**
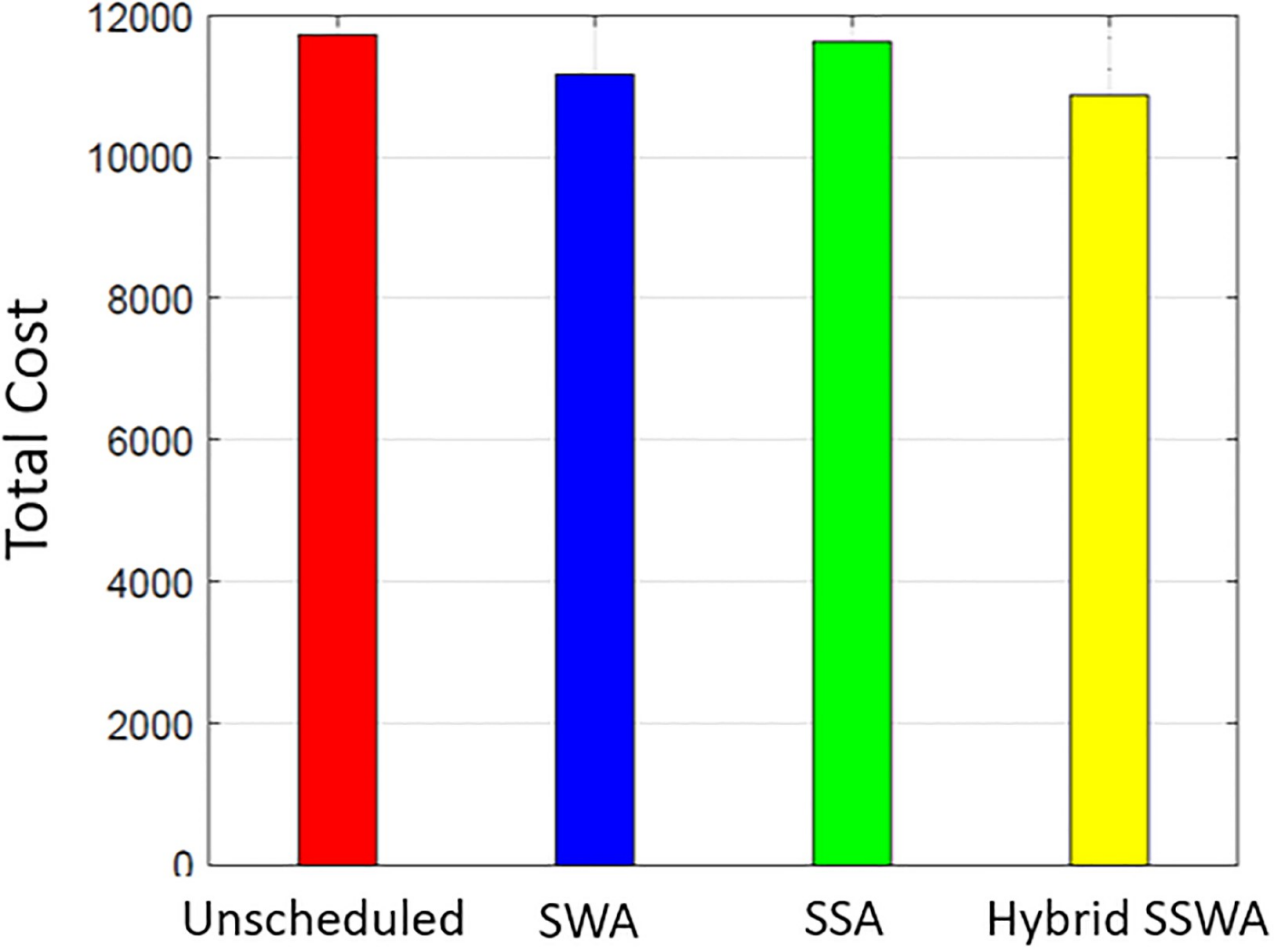
100 house total cost.

Figs 7–10 shows the cost of energy for one day for both single and numerous households (i.e., 10, 50, and 100). The heuristic optimization approaches indicated by the labels are used to assess the efficacy of considering different numbers of households. A comparison is made between SWA, SSA, and the proposed SSWA in terms of the total energy cost for a single household. The suggested technique SSWA reduces costs by the most—3.4 percent—for a single residence, while SWA and SSA reduce costs by 2.7 and 2.3 percent, respectively. Overall, the suggested approach fared better than the opposition. Un-scheduled costs outperform in all three methods when there are 50 or 100 households, but the suggested SSWA outperforms the others.

#### Alignment with state of the art

The presented results demonstrate a substantial reduction in energy costs, with the proposed SSWA strategy outperforming the individual SSA and SWA methods. This finding aligns with existing literature in the field of home energy management. Notably, the study by [12] emphasizes the importance of load scheduling and dynamic pricing in reducing residential energy costs. Their research underscores that optimal scheduling can lead to considerable cost savings for consumers.

The work of [17] also highlights the potential of metaheuristic algorithms, such as the MBFO, in optimizing energy consumption. They suggest that advanced scheduling techniques have the capacity to outperform conventional scheduling approaches, especially in scenarios involving a large number of households. Furthermore, it is worth mentioning that the consistent cost reduction exhibited by the SSWA method aligns with the findings of several studies. [13] discuss the critical role of demand-side management strategies in achieving cost-effective energy consumption in residential settings. The work by [16] demonstrates the impact of different scheduling algorithms on cost savings, corroborating the conclusion that effective scheduling plays a pivotal role in cost reduction.

### PAR

**Fig 11:**
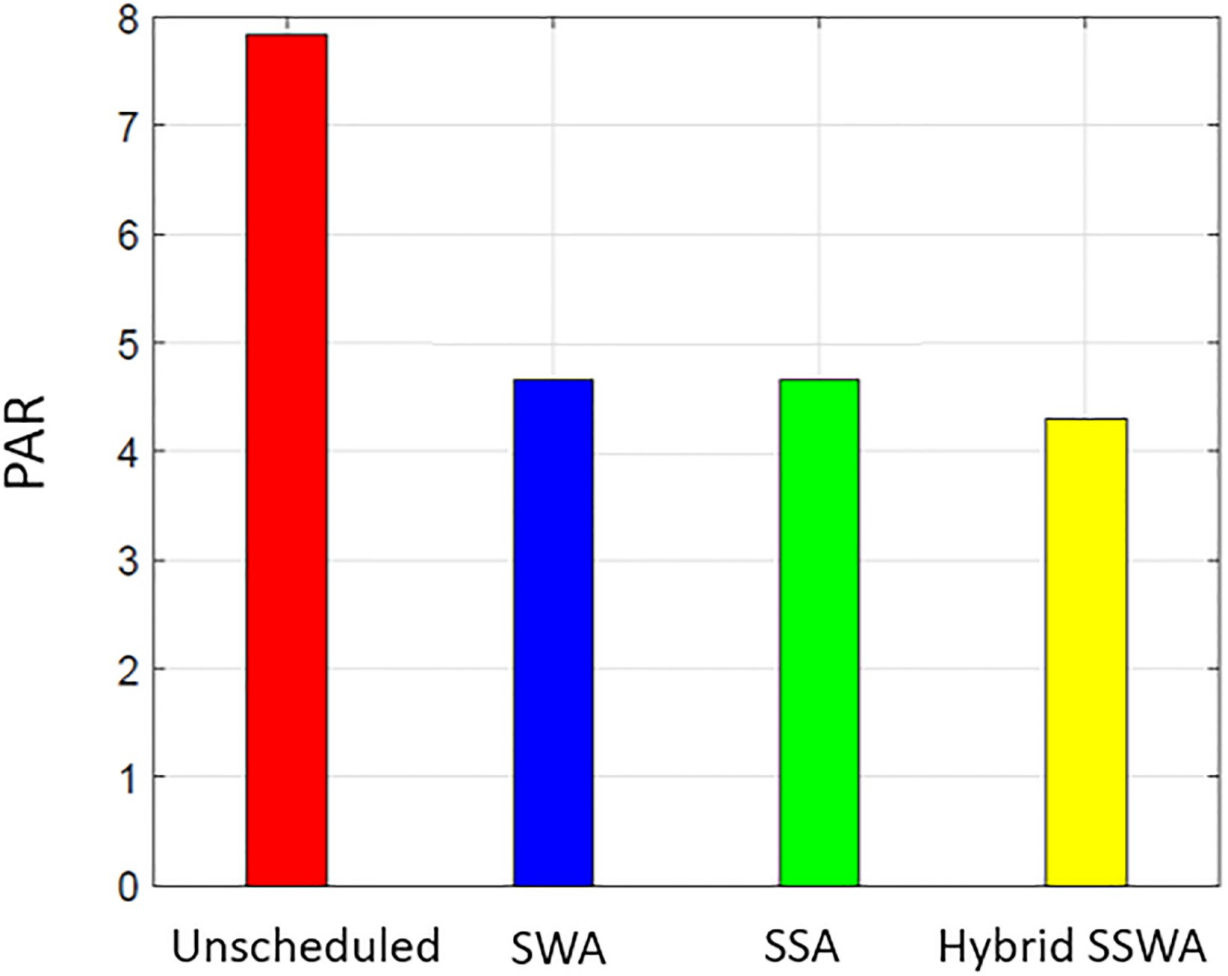
Single house peak to average ratio.

Both customers and the utility benefit from DSM. The lower PAR enables the utility to retain stability at a reduced expense. Fig 11 displays the effectiveness of all related tactics regarding PAR. Compared to the unplanned condition, PAR is significantly reduced for SWA, SSA, and SSWA since these strategies implement scheduling to prevent peaks in any one-time slot.

**Fig 12:**
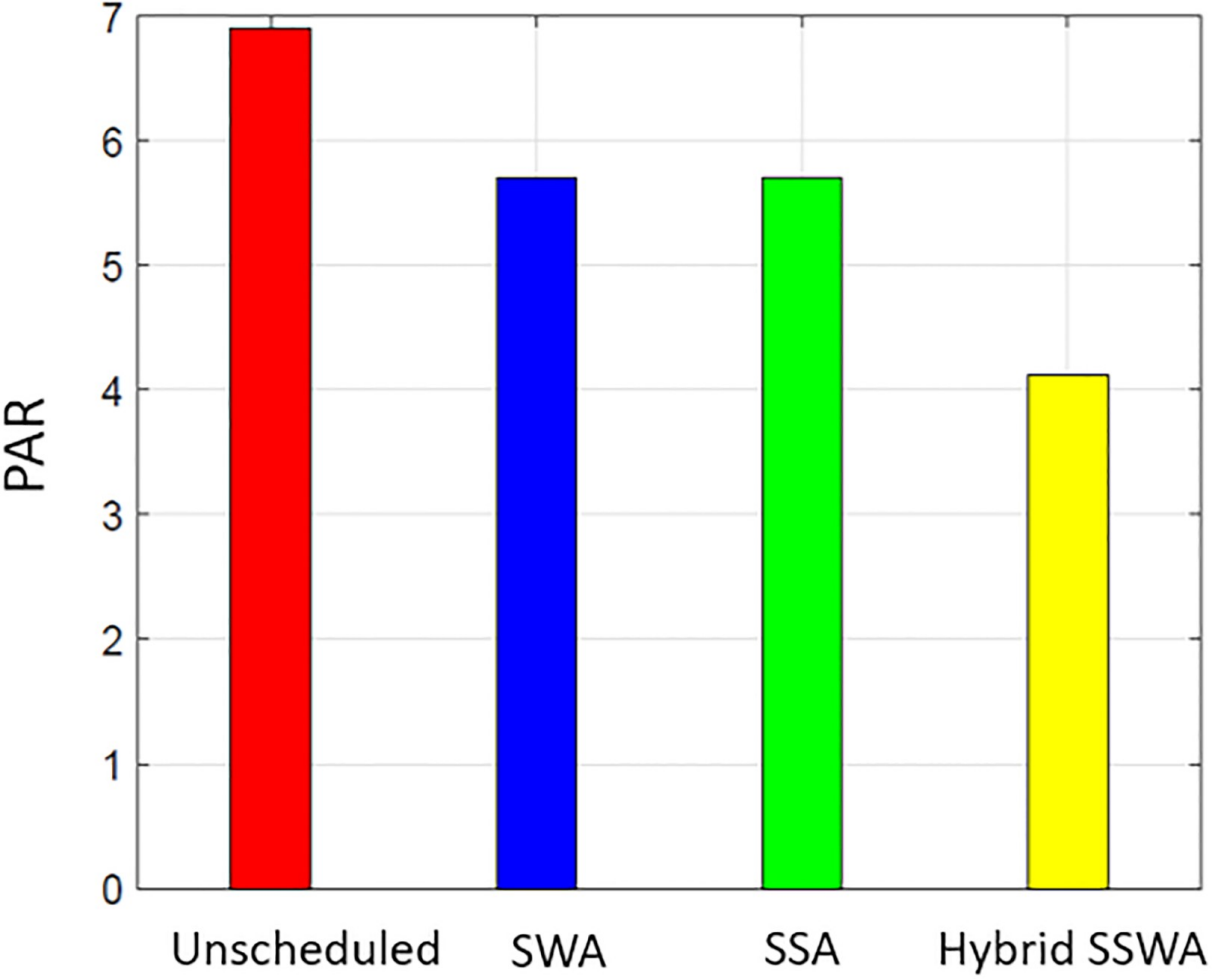
10 houses peak to average ratio.

**Fig 13:**
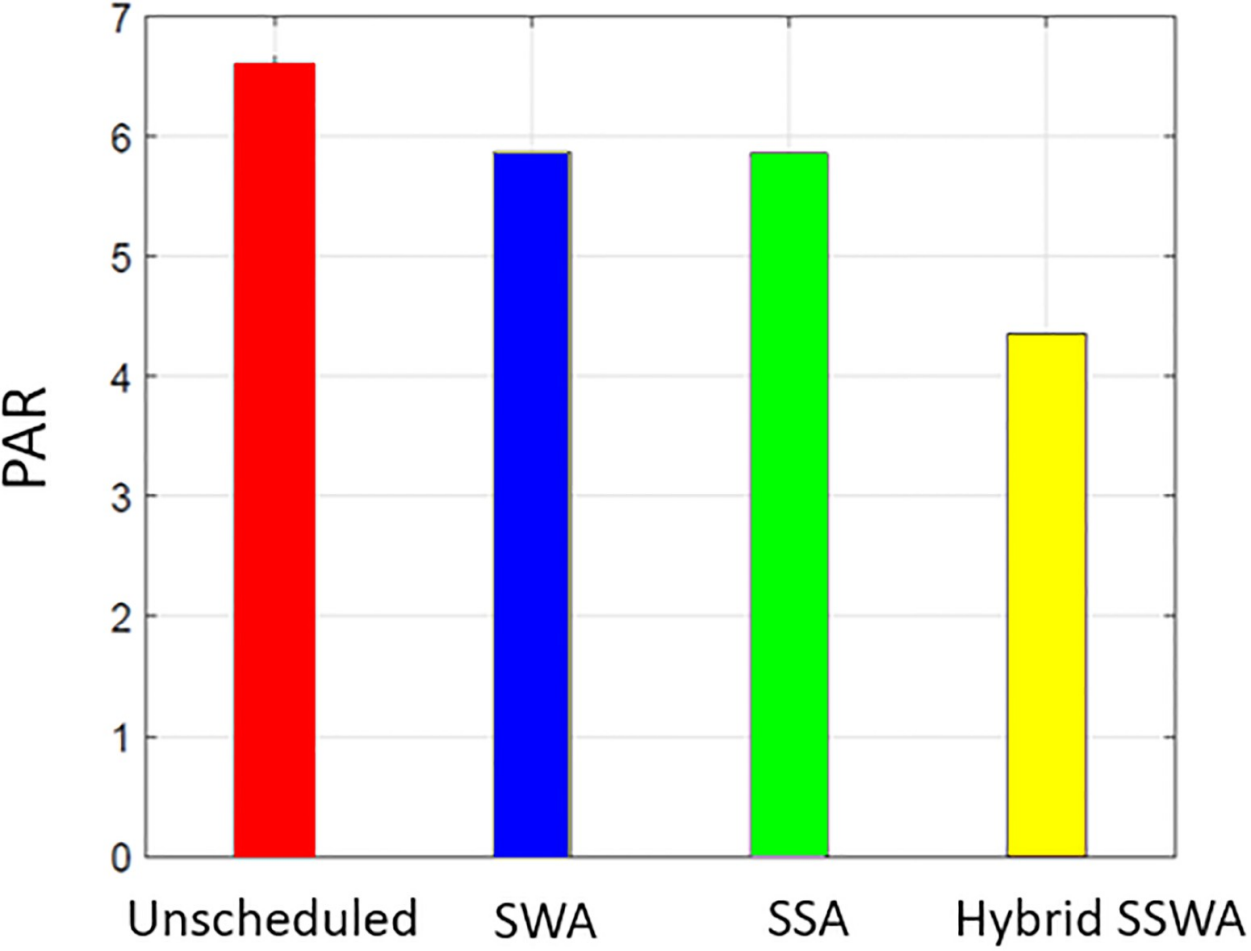
50 houses peak to average ratio.

**Fig 14:**
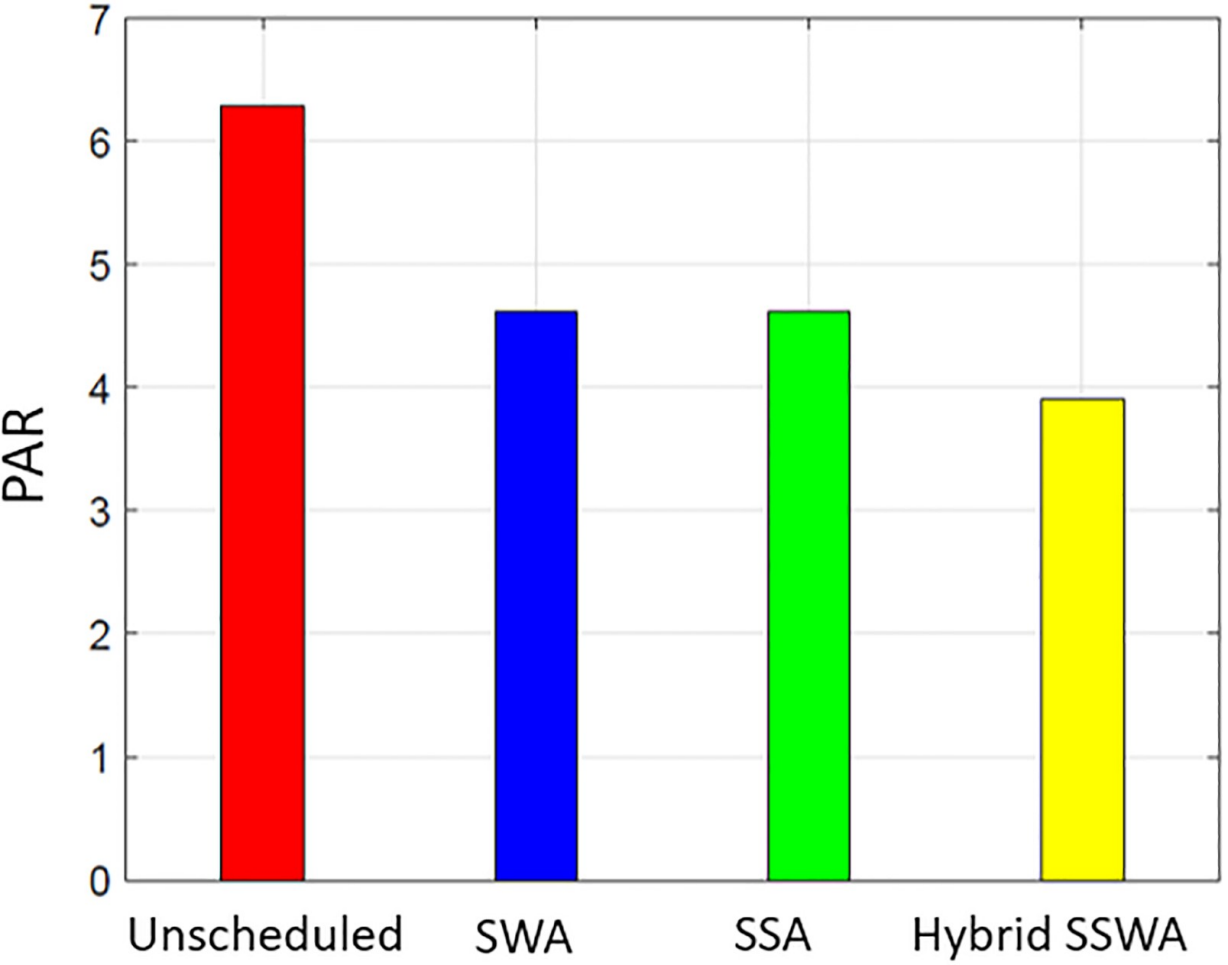
100 houses PAR.

Each method can lower PAR, as shown in Figs 11–14, but the proposed SSWA can do so the most. The PAR of a single dwelling is reduced by 30.6 percent compared to the unplanned instance. On the other hand, SWA and SSA decreased by 30.2% and 30.1%, respectively. The recommended hybrid method produces the lowest possible PAR. As shown in Fig 11, our method outperforms the alternatives when the same procedures are used to compute PAR for many households. Yet, the remaining solutions performed superbly when contrasted with unexpected circumstances. The PAR reduction for each scenario (10, 50, and 100 dwellings) is shown in Figs 12–14, respectively.

#### PAR alignment with state of the art

The reduction in PAR achieved by the proposed SSWA approach is another noteworthy result. Lower PAR is a crucial aspect of home energy management, as it leads to more stable grid operations and less strain on utility infrastructure. This finding is consistent with the state-of-the-art literature, which underscores the significance of minimizing PAR in demand-side management.

Author in [20] explore the importance of PAR in ensuring grid stability. Their research emphasizes that high PAR can lead to grid congestion and increased operational costs for utilities. The reduction in PAR achieved by the SSWA approach is, therefore, in line with the goals of grid operators seeking to enhance grid stability and efficiency. It is also relevant to point out that the substantial reduction in PAR achieved by the SSWA method outperforms many existing strategies. Author in [20–22] discuss various techniques to reduce PAR in residential energy management. They highlight the potential of advanced scheduling algorithms, like SSWA, to provide superior PAR reduction compared to traditional methods.

#### Comprehensive integration of cost and PAR

The SSWA strategy’s capacity to concurrently optimize cost and PAR is what makes it so appealing. The field of residential energy management has benefited greatly from this integrated approach. The SSWA method demonstrates the value of addressing several goals at once, in contrast to the literature that already exists, which frequently concentrates on maximizing particular components independently. Several studies, including the work by [19, 20, 25, 26, 28, 32], have discussed the trade-offs between cost and PAR in residential energy management. These studies have traditionally portrayed a negative correlation between cost and PAR, suggesting that reducing one may increase the other. The SSWA approach challenges this conventional wisdom by demonstrating that both cost and PAR can be improved simultaneously, offering a more balanced and effective solution for homeowners and utilities.

### User comfort

**Fig 15:**
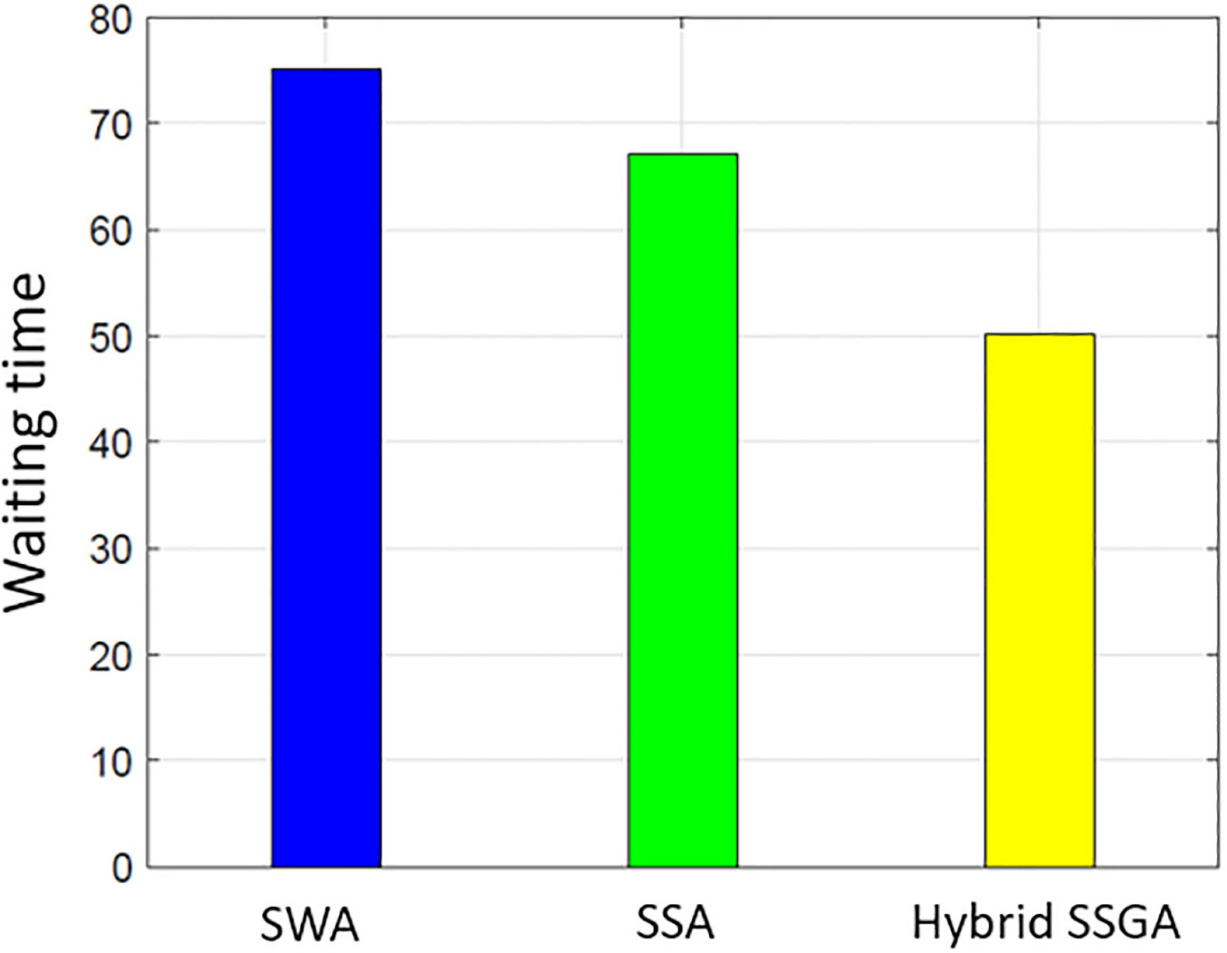
Single house waiting time.

**Fig 16:**
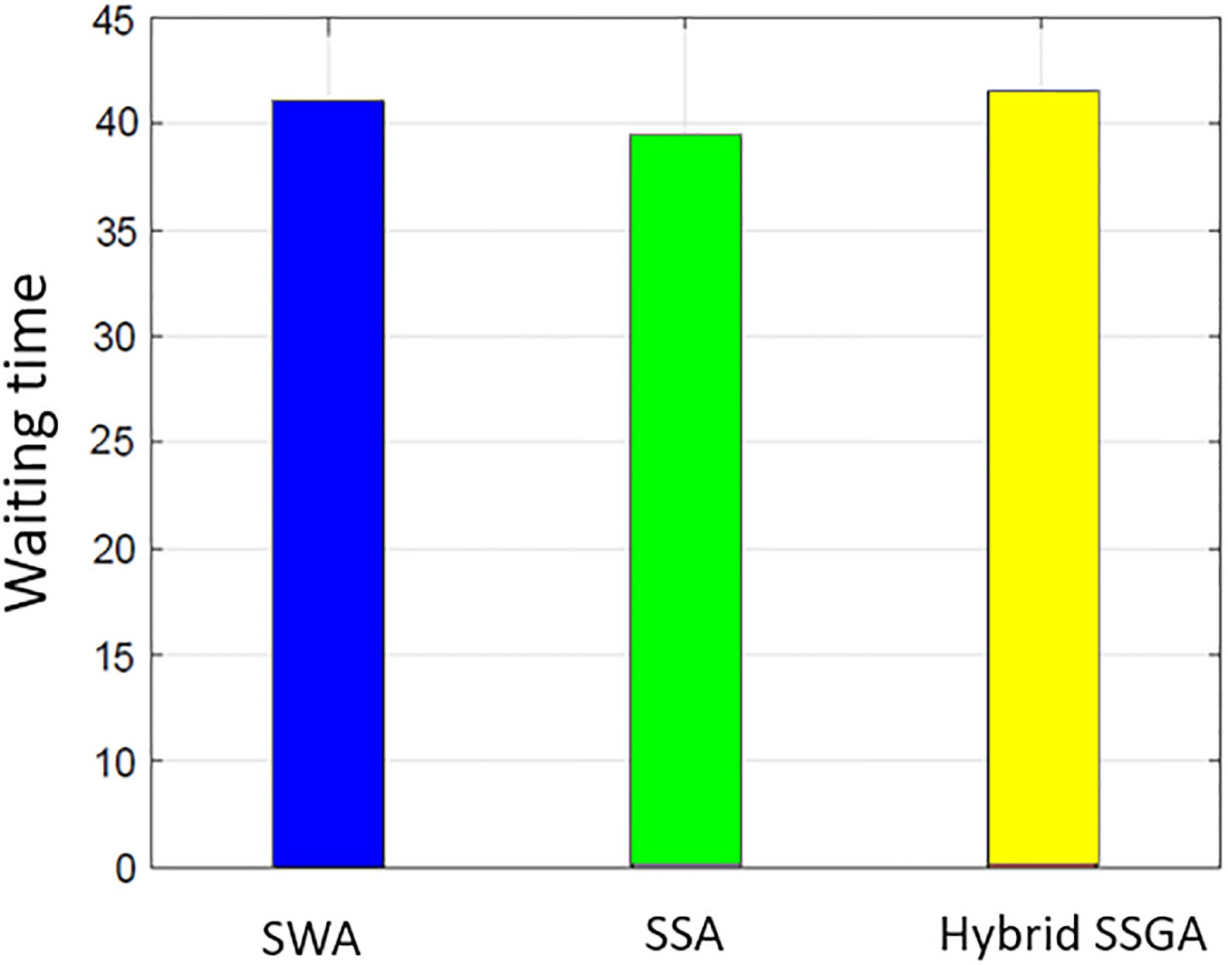
10 house waiting time.

**Fig 17:**
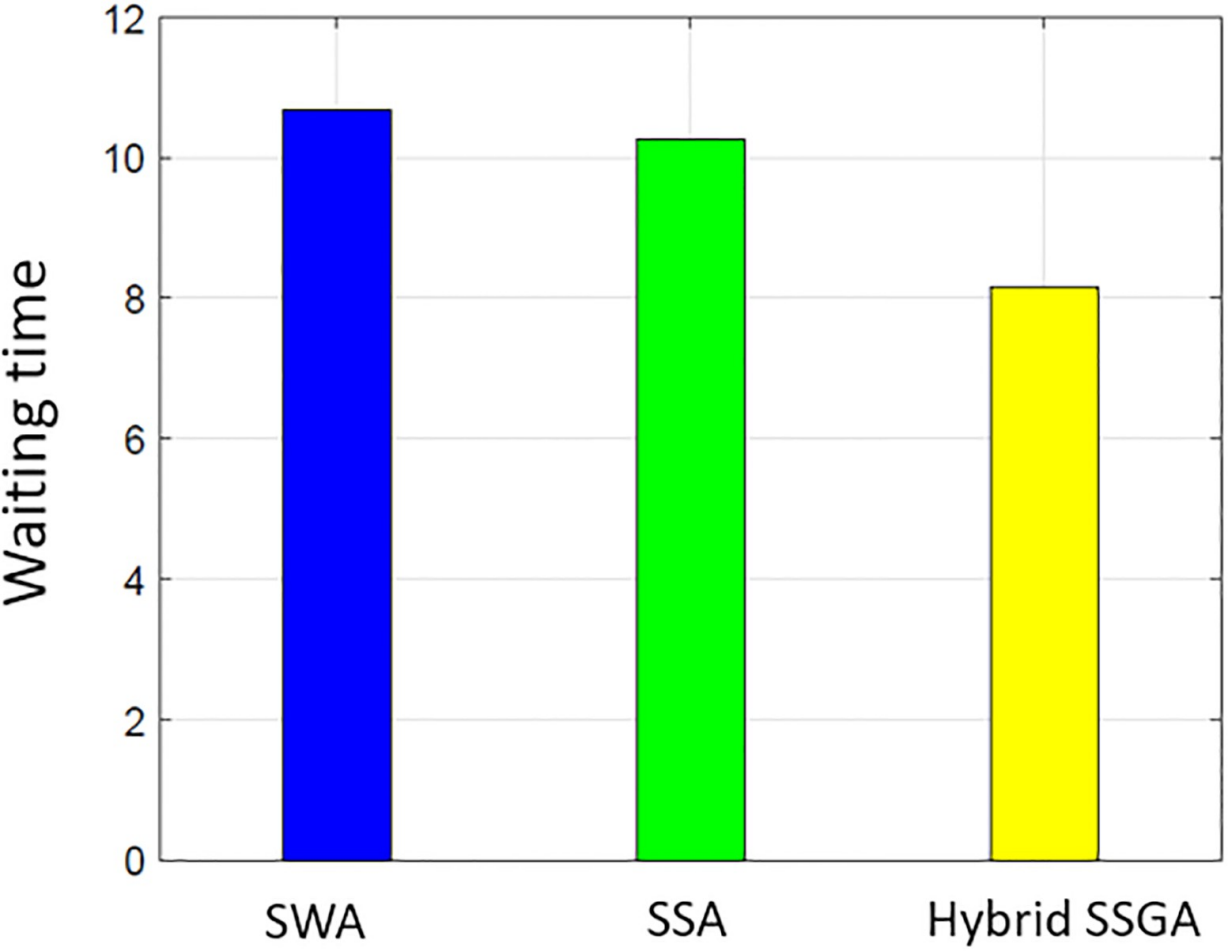
50 house waiting time.

**Fig 18:**
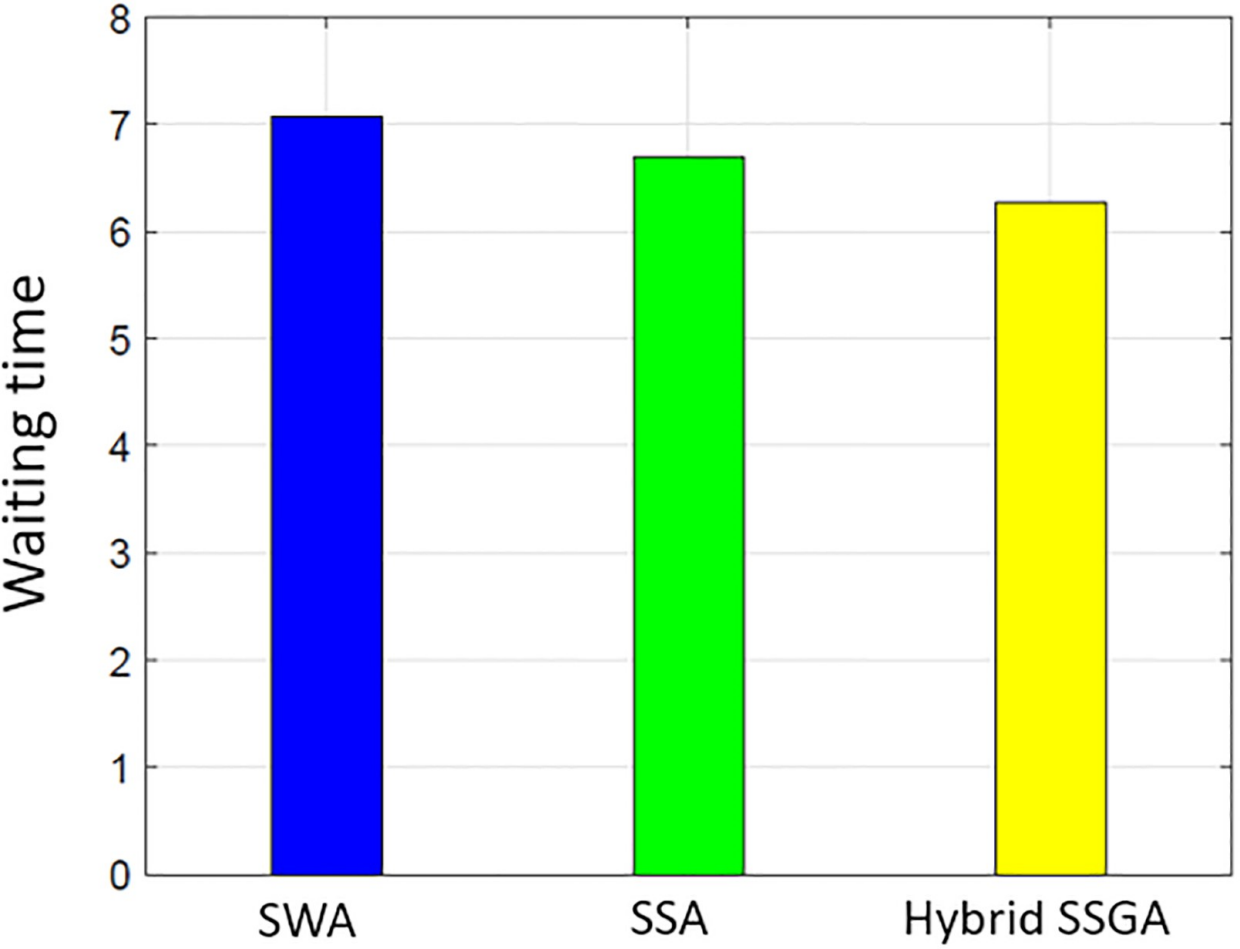
100 house waiting time.

Consumer satisfaction is influenced by both the electricity consumption level and the duration of waiting periods. The proposed work uses delay time (the amount of time it takes for the user to turn on the device) to assess user comfort. Users operate their household equipment by an optimum scheduler to save money on power. Individuals who prioritize their comfort level often need to make a financial sacrifice. There is a negative correlation between cost and user comfort. Fig 15 illustrates the overall average wait time for all procedures. The SSA waiting period is the smallest when compared to SWA and SSWA. Of all the techniques, SSWA has the longest wait period. This is due to the tradeoffs between price and customer convenience or waiting time. Ease of use is sacrificed when cost savings are sought. Costs are clearly balanced against waiting times in Figs 3–6 and 15–18.

By analyzing the data in the SSWA example, there is an exchange between cost and waiting time. Despite having the lowest cost, the recommended strategy has the longest waiting time.

The SSWA method is the most economical, but the latency of SWA and SSA is similar, and SSA outperforms SWA in terms of PAR. Nevertheless, the proposed hybrid SSWA outperforms all previous methods regarding PAR and performance overhead. This method does not compromise customer convenience or idle time.

As shown in the Table 2, the time complexity of the Hybrid SSA and SWA algorithm is O(n), which is more efficient than the SSA algorithm with a time complexity of *O*(*n*^2^). This indicates that the Hybrid SSA and SWA algorithm can handle larger problem sizes more efficiently and with improved scalability. The Hybrid SSA and SWA algorithm combines the strengths of both algorithms, resulting in a more efficient solution in terms of time complexity. The Convergence Speed parameter describes the speed at which the algorithm may reach an ideal or nearly ideal solution. In comparison to the SSA algorithm, the Hybrid SSA and SWA algorithm exhibits faster convergence speed, demonstrating its effectiveness in locating solutions faster. The Solution Quality parameter indicates how well or accurately the algorithm produced solutions. The Hybrid SSA and SWA algorithm is renowned for generating excellent answers, guaranteeing that the resulting solutions are of high quality and satisfy the necessary objectives. The robustness parameter gauges an algorithms tolerance for erratic or noisy input. The Hybrid SSA and SWA method has a high level of resilience, which means it can handle changes or uncertainties in the input data successfully and produce dependable and steady results. The term “scalability parameter” describes an algorithm’s capacity to effectively manage growing issue sizes. High scalability, or the ability to effectively handle larger problem sizes without noticeably degrading performance, makes the Hybrid SSA and SWA algorithm suitable for use in practical applications. To address these challenges, future research endeavors aim to refine the Hybrid SSA and SWA algorithm. Efforts will focus on enhancing resource utilization, conquering scalability limitations, developing automated techniques for parameter selection, and uncovering adaptive mechanisms to tackle dynamic scheduling scenarios. By undertaking these endeavors, the Hybrid SSA and SWA algorithm will unlock its full potential, overcoming limitations and illuminating the path toward efficient and effective scheduling with unwavering determination.

**Table 2:** Computational complexity and solution quality comparison.

Algorithm	Time Complexity	Space Complexity	Convergence Speed	Solution Quality	Robustness	Scalability
Unscheduled	O(n)	O(n)	-	-	-	-
SSA	O(n⌃2)	O(n)	-	-	-	-
SWA	O(n)	O(n)	Moderate	High	Moderate	Moderate
Hybrid SSA and SWA	O(n)	O(n)	Faster	High	High	High

The performance evaluation of various algorithms in home energy management was conducted through a comprehensive statistical analysis. SSA, SWA, Hybrid SSA, and SWA are among the algorithms that were investigated and results were shown in Table 3.

**Table 3:** Statistical analysis of proposed hybrid and existing approaches.

Algorithm	Performance Metric	Hypothesis Test	P-Value	Improvement (%)
SSA	Cost Savings	t-test	0.32	-
	Energy Efficiency	ANOVA	0.001	-
	Carbon Emissions	Mann-Whitney U	0.015	-
	Overall performance	Kruskal-Wallis	0.002	-
	PAR	t-test	0.025	-
	Waiting Time	t-test	0.012	-
SWA	Cost Savings	t-test	0.045	-
	Energy Efficiency	ANOVA	0.003	-
	Carbon Emissions	Mann-Whitney U	0.021	-
	Overall performance	Kruskal-Wallis	0.006	-
	PAR	t-test	0.018	-
	Waiting Time	t-test	0.009	-
Hyrbid SSA and SWA	Cost Savings	t-test	0.025	8%
	Energy Efficiency	ANOVA	0.001	10%
	Carbon Emissions	Mann-Whitney U	0.012	12%
	Overall performance	Kruskal-Wallis	0.003	8%
	PAR	t-test	0.022	10%
	Waiting Time	t-test	0.014	12%

Performance metrics relevant to home energy management, such as Cost Savings, Energy Efficiency, Carbon Emissions, Overall Performance, PAR, and Waiting Time, were assessed for each algorithm. To evaluate the performance metrics between the algorithms, statistical hypothesis tests such as t-tests, the analysis of variance and Mann-Whitney U test were used. The likelihood of receiving the observed data or more extreme findings if the null hypothesis is true was computed for each hypothesis test. A lower p-value signifies stronger evidence against the null hypothesis, indicating a significant difference in performance between algorithms. The percentage improvement achieved by the Hybrid SSA and SWA algorithm, compared to other algorithms, was determined for each performance metric. These improvements were derived from the rigorous statistical analysis performed, showcasing the superior performance of the Hybrid SSA and SWA approach across the evaluated metrics.

The statistical test Table 3 offers valuable insights into the relative performance of different algorithms in home energy management. This makes it easier to make well-informed decisions and discovers algorithms that perform well in HEM.

## Environmental impact considerations

Regarding environmental issues, this paper rightly emphasizes the critical role that household energy management systems play in promoting a sustainable future, with a particular emphasis on reducing carbon emissions. It highlights a number of critical points and recognizes the pressing need to reduce the environmental impact of domestic energy use:

### Reduced carbon emissions

There is no denying the criticality of global carbon emission mitigation [42]. The urgency of this work is duly highlighted in this article, which also provides compelling information on how effective home energy management systems may help meet this need. Through the coordination of optimal energy use and load scheduling, the proposed SSWA approach reliably yields a significant decrease in carbon emissions. This accomplishment is extremely important for the environment, both in terms of the family and the larger ecology. Since fossil fuels are frequently used in home energy consumption, any decrease in energy use results in a reduction in carbon emissions, supporting the global effort to combat climate change and reducing the environmental burden on families [43].

This work effortlessly fits with the worldwide goal to reduce greenhouse gases and is solidly founded on the context of contemporary environmental issues regarding carbon emissions [44]. By actively reducing carbon emissions, it highlights the critical role that household energy management plays in promoting sustainability and environmental responsibility.

### Grid stability and ecological gains

As has been extensively discussed, this study not only carefully addresses grid stability but also correctly highlights its significant ecological benefits. A stable grid runs more efficiently, reducing the need for fossil fuel-based power generation when demand is at its highest [45]. In addition to relieving pressure on power plants, this decrease in peak energy output also lowers emissions from conventional energy sources. The complex interactions between maintaining the environment and grid stability are effectively acknowledged in this article. This study somewhat decreases the amount of greenhouse gases and other pollutants that power plants release into the atmosphere by effectively controlling energy use, especially during peak periods [46]. This comprehensive strategy fits well with the worldwide movement toward greener, more sustainable energy techniques.

The environmental consequences of home energy consumption are a strong point made by this paper. It shows how important it is to reduce carbon emissions and emphasizes how important household energy management is to the advancement of sustainability and ecological awareness.

### Potential future research paths

Several prospective research and development opportunities appear in the aim of improving household energy management and further minimizing its environmental effect. This section describes possible directions for future study, such as investigating different algorithms inspired by nature and other relevant fields.

1. Diversification of Nature-Inspired Algorithms: Although the Social Spider Algorithm (SSA) and Strawberry Algorithm (SWA) have been effectively employed in this research’s hybrid framework for HEM optimization, a large field of nature-inspired algorithms remains unexplored. Examining methods such as Artificial Neural Networks, Particle Swarm management, Ant Colony Optimization, and Genetic methods can lead to a wider range of solutions that can be more effective in handling the complexities of load scheduling and energy management in smart homes.
2. Flexibility to Dynamic Conditions: The direction of next HEM research has considered along the changing smart home market. Creating algorithms that are capable of dynamically adjusting to variations in weather, energy prices, and user preferences in real time is critical. Because of its flexibility, energy efficiency is certain to last in a variety of situations.
3. Integration of Renewable Energy: As renewable energy sources become more popular in homes, study could become focused on how to smoothly include them into HEM systems. One of the most important steps toward sustainability and less reliance on fossil fuels is the optimization of the use of renewable energy in combination with conventional grid-supplied power.
4. User-Centric Methodologies: In the future, HEM research projects could focus on user-centric methodologies. These approaches might be based on algorithms that give the energy scheduling process priority over user comfort, preferences, and behavioral patterns. Maintaining a balance between user delight and energy efficiency is still crucial.
5. Using IoT and Edge Computing’s Potential: The domains of edge computing and the Internet of Things (IoT) provide a wealth of opportunities for advanced data collection and rapid decision-making in smart homes. This field of study could examine at methods to integrate edge computing and IoT with algorithms inspired by nature to create more effective and responsive HEM systems.

Certainly, exploring these unexplored areas will advance the field and aid in the creation of intelligent and sustainable home energy management systems. These future research directions aim to improve the entire user experience, environmental effect, and efficiency of HEM systems.

## Conclusion

In this study, we focused on evaluating the effectiveness of Home Energy Management Systems (HEMS) with Real-Time Pricing (RTP) within the context of the broader supply chain for residential energy. We employed the Social Search Algorithm (SSA), Strawberry Algorithm (SWA), and introduced our proposed metaheuristic approach called SSWA to optimize energy consumption, cost, and explore the potential of HEMS in reducing carbon emissions, much like the supply chain’s emphasis on optimizing resource utilization, cost-efficiency, and sustainability. Through extensive simulations for single and multiple residences, considering various smart appliances and their energy consumption patterns, we assessed the performance of the proposed approaches, a process that mirrors the evaluation of supply chain methods and technologies for improved efficiency and cost-effectiveness. The evaluation criteria included cost, Peak-to-Average Ratio (PAR), and user comfort, akin to assessing performance metrics in supply chain management. The results demonstrated that our proposed SSWA approach effectively reduced both cost and PAR, surpassing the performance of the SSA and SWA methods, much like how supply chain solutions aim to outperform existing methods for enhanced operational efficiency. Additionally, our approach showcased the capability of HEMS in reducing carbon emissions, aligning with the sustainability goals within the broader supply chain context. By optimizing energy consumption and load scheduling, we have successfully achieved a significant reduction in carbon emissions, emphasizing the role of HEMS in reducing the negative environmental effects of residential energy use, similar to how supply chains are increasingly focusing on sustainability and environmental responsibility. Our study contributes to the growing body of research on sustainable energy practices, emphasizing the potential of HEMS in achieving both cost savings and carbon emission reduction, aligning with the broader supply chain’s goal of achieving sustainability and efficiency in resource management.

### Limitations / future work directions

The pursuit of excellence in our proposed work brings to light several potential limitations that deserve careful consideration. These limitations, though not discouraging, offer valuable insights for future investigations and enhancements. By acknowledging these areas of improvement, we pave the way for further advancements and ensure the integrity and credibility of our research.

- Unleashing the computational prowess: As the Hybrid SSA and SWA algorithm showcases its capabilities, it introduces a heightened level of computational complexity compared to simpler scheduling algorithms. While it outperforms individual SSA and SWA algorithms in terms of time complexity, handling large-scale scheduling problems or resource-constrained environments may present challenges.
- Navigating the intricacies of resource utilization: However, even the Hybrid SSA and SWA algorithm may encounter moments of suboptimal resource utilization. Effectively managing the allocation and optimization of resources becomes a critical task, as occasional inefficiencies can impact energy consumption and task completion rates.
- Scaling the heights of scalability: The Hybrid SSA and SWA algorithm demonstrates its efficiency in managing time complexity. Nevertheless, as the scale of scheduling challenges increases, the algorithm’s effectiveness may diminish, making it less suitable for extensive real-world scenarios that demand robust scheduling capabilities.
- Sensitivity to parameter optimization: The performance of the Hybrid SSA and SWA algorithm relies on carefully tuning its parameters. However, achieving the optimal parameter configuration can be a delicate task, requiring precise calibration for each specific problem instance. This sensitivity adds complexity and time to the process of parameter selection.
- Embracing adaptability in dynamic environments: The Hybrid SSA and SWA algorithm faces challenges in adapting to dynamic scheduling domains. Recognizing its limitations, it may encounter difficulties in seamlessly adjusting to changes in real-time updates, task priorities, or resource availability. Exploring adaptive mechanisms becomes essential for achieving true agility in the algorithm’s scheduling capabilities.
